# A microbiota–metabolite–glial axis drives white matter repair via isorhamnetin–PSAP–GPR37 signaling after ischemic stroke

**DOI:** 10.1080/19490976.2026.2748092

**Published:** 2026-11-01

**Authors:** Yue Zhang, Lina Qiu, Jiajun Wu, Yao Shi, Shenna Niu, Chengcheng Zhao, Tao Chen, Chengcheng Cui, Yilin Ma, Xuebin Zhang, Xiuhua Yao, Yaping Meng, Lingmin Zhao, Yuxin Xing, Xiaojuan Wu, Zhili Chen, Kai Zheng, Ying Cai, Jieli Chen, Jialing Wu

**Affiliations:** a Huanhu Hospital Affiliated to Tianjin Medical University, Tianjin, China; b Tianjin Key Laboratory of Cerebral Vascular and Neurodegenerative Diseases, Tianjin, China; c Department of Neurology, Tianjin Huanhu Hospital, Tianjin, China; d Huanhu Hospital, Tianjin University, Tianjin, China; e Tianjin Neurosurgical Institute, Tianjin Huanhu Hospital, Tianjin, China; f Gerontology and Neurological Institute, Tianjin Medical University General Hospital, Tianjin, China

**Keywords:** Ischemic stroke, white matter repair, microbiota, metabolite, microglia, oligodendrocytes

## Abstract

Ischemic stroke (IS) induces persistent neuroinflammation and white matter injury that limit neurological recovery, yet effective repair-oriented therapies remain lacking. Although microbiota influences stroke outcomes, how microbial activity drives central nervous system repair is unclear. Here, we integrate clinical microbiome profiling, experimental stroke models, multi-omics analyses, and mechanistic validation to define a microbiota–metabolite–glial signaling axis that promotes post-stroke recovery. *Akkermansia muciniphila* (Akk) is associated with favorable outcomes in patients and improves recovery in mice without altering infarct size, supporting a repair-promoting rather than neuroprotective mechanism. Mechanistically, Akk increases circulating isorhamnetin (Iso), a key systemic mediator that suppresses lipid-laden, pro-inflammatory microglia and promotes oligodendrocyte maturation and white matter repair. Iso reprograms microglia to secrete PSAP, and conditioned medium-but not direct Iso-activates GPR37–ERK signaling in oligodendrocytes. *In vitro* knockdown of *Psap* or *Gpr37* markedly attenuates these effects, demonstrating a microglia-dependent mechanism. *In vivo* adeno-associated virus (AAV)-mediated *Gpr37* knockdown abolishes the beneficial effects of Akk and Iso on functional recovery and white matter repair, establishing the necessity of PSAP–GPR37 signaling. These findings identify a microbiota-driven metabolic pathway linking gut activity to white matter repair after IS.

## Introduction

Ischemic stroke (IS) remains a leading cause of long-term disability and mortality worldwide.^1^ Although acute neuronal loss is largely determined within hours after vascular occlusion, sustained neurological impairment is increasingly attributed to chronic neuroinflammation, progressive white matter degeneration, and failure of endogenous repair mechanisms.^2^ In particular, persistent disruption of oligodendrocyte homeostasis and myelin integrity critically limits functional recovery,^3^ yet therapeutic strategies that effectively promote white matter repair after IS remain lacking.

Beyond the central nervous system, IS induces systemic alterations that extend to peripheral organs, including intestinal barrier dysfunction and gut microbiota dysbiosis. Clinical studies have shown that patients with IS exhibit marked shifts in gut microbial composition, characterized by enrichment of potentially pathogenic taxa (e.g., *Streptococcus*, *Lactobacillus*, *Escherichia*) and depletion of beneficial commensals (e.g., *Eubacterium*, *Roseburia*).^^4^^ Experimental studies further demonstrate that microbiota composition and metabolic capacity influence post-stroke inflammation and recovery; for example, transplantation of youthful or short-chain fatty acid-enriched microbiota improves neurological outcomes through immune modulation across the gut-brain axis.^5-7^ Together, these findings suggest that the gut microbiota contributes to stroke recovery. However, the specific microbial determinants and molecular pathways by which intestinal dysbiosis is transduced into glial responses and white matter repair after stroke remain poorly defined.

*Akkermansia muciniphila* (Akk) is a mucin-degrading commensal bacterium that plays an important role in maintaining intestinal barrier integrity and metabolic homeostasis.^8^ Akk abundance is reduced in inflammatory and metabolic disorders,^9^ and its supplementation has been shown to restore epithelial barrier function, suppress aberrant inflammation, and improves metabolic parameters.^10^ Emerging evidence also implicates Akk in neurological diseases: Akk supplementation alleviates disease progression in amyotrophic lateral sclerosis through modulation of host metabolism,^11^ correlates inversely with amyloid burden in Alzheimer’s disease,^12^ and improves cognitive function via microbiota- derived metabolites.^13^ In the context of IS, experimental studies suggest that higher Akk abundance is associated with improved outcomes, potentially through attenuation of neuroinflammation.^14^ ^,^ ^15^ However, whether Akk directly regulates post-stroke repair processes, particularly those involving glial lipid metabolism and white matter integrity, and the underlying molecular mechanisms remain unclear.

Here, we integrate clinical microbiome profiling, experimental stroke models, multi-omics analyses, and mechanistic validation to investigate the role of Akk in post-stroke recovery. We show that Akk supplementation promotes functional recovery after IS and is associated with increased circulating levels of the flavonoid metabolite isorhamnetin (Iso). Mechanistically, Iso suppresses lipid-laden, pro-inflammatory microglial states and promotes oligodendrocyte maturation. Further analyses identify a prosaposin (PSAP)–G-protein coupled receptor 37 (GPR37)–extracellular signal-regulated kinase (ERK) signaling axis mediating microglia–oligodendrocyte communication in this process. Together, our findings support a microbiota–metabolite–glia signaling pathway that links gut microbial activity to white matter repair after ischemic stroke and suggest a potential strategy for enhancing post-stroke recovery.

## Methods

### Human participants and sample collection

Sample size estimation was based on the anticipated association between changes in National Institutes of Health Stroke Scale (NIHSS) scores and the exposure variable. Assuming a correlation coefficient of 0.25, a two-sided significance level of 0.05, and 80% statistical power, the estimated minimum sample size was approximately 123 patients with ischemic stroke. Allowing for potential attrition due to loss to follow-up, assay failure, or missing data, we initially aimed to recruit 140–150 patients. However, because of constraints in clinical recruitment and the availability of eligible fecal samples, the prespecified recruitment target was not reached. Therefore, the clinical analyses in the present study were considered exploratory.

A total of 114 participants were ultimately enrolled, comprising 86 patients with ischemic stroke admitted to Tianjin Huanhu Hospital and 28 healthy controls. The study involving human participants was conducted in accordance with the Declaration of Helsinki and was approved by the Ethics Committee of Huanhu Hospital Affiliated to Tianjin Medical University (approval number 2024–043). Written informed consent to participate was obtained from all participants before enrollment. For participants who were unable to provide informed consent themselves, written informed consent was obtained from their legally authorized representatives. No minors were enrolled in the study.

Inclusion criteria for IS patients were as follows: (i) a diagnosis of ischemic stroke confirmed by focal neurological deficits and magnetic resonance imaging (MRI); (ii) age between 18 and 80 y; and (iii) hospital admission within 7 d of stroke onset. Healthy controls had no history of cerebrovascular, gastrointestinal disease, or malignancy.

Exclusion criteria applied to all participants: (i) any history of gastrointestinal surgery or pre-existing gastrointestinal diseases; (ii) use of antibiotics or probiotics within 1 month prior to enrollment; (iii) unstable vital signs; (iv) special dietary habits (e.g., vegetarianism). Fresh fecal samples were collected from all participants and immediately stored at −80 °C for 16S rDNA sequencing. For IS patients, fecal samples were obtained within 7 d after stroke onset. Demographic characteristics and relevant clinical data were recorded.

For stroke patients, neurological impairment was quantified using the NIHSS. Early short-term functional prognosis was quantified by calculating the difference between admission NIHSS score and discharge NIHSS score. Patients whose discharge NIHSS score was lower than their admission NIHSS score were categorized into the favorable prognosis group, while the rest were assigned to the other outcome group.

### Animals, photothrombotic middle cerebral artery occlusion (pMCAO) model establishment, and experimental design

Animals: Male C57BL/6 mice (6–8 months old) were obtained from SPF Biotechnology Co., Ltd. (China) and housed at Tianjin Huanhu Hospital under specific pathogen-free conditions (23 °C, 50% humidity, 12-hour light/dark cycle) with ad libitum access to food and water. Mice were allowed to acclimate for 7 d before experimentation. All animal procedures complied with the Guide for the Care and Use of Laboratory Animals and were approved by the Animal Experiments Ethical Committee of Tianjin Medical University (Ethical No. HHYY2025–010). No formal a priori sample-size calculation was performed for the animal experiments. Sample sizes were determined based on previous studies using comparable experimental paradigms.

#### pMCAO model

Focal cerebral ischemia was induced using the pMCAO model. Mice were anesthetized with isoflurane (3% for induction and 1.5% for maintenance) delivered in oxygen through a nose cone and positioned in a stereotaxic frame. Adequate anesthetic depth was confirmed by the absence of the pedal withdrawal reflex. Body temperature was continuously monitored and maintained at 37 ± 0.5 °C using a temperature-controlled heating pad throughout the procedure. Rose Bengal (100 μg/g body weight; Sigma-Aldrich, USA) dissolved in sterile saline was administered via intraperitoneal injection 5 min before illumination. Following scalp incision, the skull was exposed and illuminated with a cold green light source (535 ± 25 nm; KL1600 LED, SCHOTT, Germany) positioned 2 mm lateral to bregma, targeting the sensorimotor cortex according to the Franklin and Paxinos mouse brain atlas. Illumination was applied for 20 min. After illumination, the incision was sutured, and mice were allowed to recover on a temperature-controlled heating pad. Sham-operated mice received Rose Bengal injection without illumination. Meloxicam (5 mg/kg) was administered subcutaneously once every 24 h for 2 d after the surgery as post-operative care for pain relief.

Neurological deficits were assessed at 24 h after surgery using the modified neurological severity score (mNSS). Mice with mNSS scores ranging from 6 to 12 at 24 h after photothrombotic stroke induction were included in subsequent experiments.

#### Experimental design and treatments

Akk treatment groups: *Akkermansia muciniphila* (live bacteria; quantified as active fluorescent units, AFU; Pendulum Therapeutics, USA) was resuspended in sterile phosphate-buffered saline (PBS) and administered by oral gavage once daily for 14 consecutive days. Vehicle-treated sham and pMCAO mice received 200 μL PBS by oral gavage.

Mice were randomly assigned to four experimental groups (Sample sizes for each experiment are indicated in the corresponding figure legends):

(1) Sham group;
(2) pMCAO + vehicle group (phosphate-buffered saline, PBS);
(3) pMCAO + low-dose Akk group (L-Akk, 4 × 10^6^ AFU);
(4) pMCAO + high-dose Akk group (H-Akk, 8 × 10^6^ AFU).

Iso and PSAP treatment: To evaluate the effects of the microbiota-regulated metabolite isorhamnetin (Iso) and the pathway effector prosaposin (PSAP), a separate cohort of pMCAO mice was used. Mice received PBS, Iso (5 mg/kg; HY-N0776, MedChemExpress, USA; intraperitoneal injection), or PSAP (9 μg/kg; HY-P77159, MedChemExpress, USA; intranasal administration) at 24 h after surgery. The dosing regimen was selected based on prior studies demonstrating efficacy in MCAO models.^16^ ^,^ ^17^ Mice were euthanized at day 14 using the procedure described.

Randomization and blinding: Animals were randomly assigned to either the sham or stroke groups and received treatments determined by a lottery drawing method. Investigators conducting behavioral assessments, histological analyses, and molecular measurements were blinded to group assignments throughout data acquisition and analysis.

### Animal welfare monitoring and humane endpoints

Animals were monitored daily after surgery for general appearance, body weight, spontaneous activity, grooming, food and water intake, mobility, and signs of pain or distress. Predefined humane endpoints were established in accordance with the approved institutional animal protocol. Predefined humane endpoints included excessive 20% body-weight loss, inability to eat or drink, persistent recumbency or inability to ambulate, severe neurological deterioration, or other signs of severe and unrelieved pain or distress. Animals reaching any humane endpoint were immediately euthanized. All animal monitoring and welfare procedures were conducted in accordance with the approved institutional animal protocol (Ethical No. HHYY2025–010).

### Neurological and cognitive behavioral assessments

Neurological and cognitive behavioral tests were performed to assess functional recovery after pMCAO. Neurological functions were evaluated at baseline (before pMCAO) and at day 1, 3, 5, 7, 10, and 14 after pMCAO, and cognitive behavioral assessments were assessed at day 14 after pMCAO. All tests were conducted by investigators blinded to group allocation, with three repeated measurements per session, and the average value was used for analysis.

mNSS: Neurological deficits were assessed using the mNSS, which evaluates motor function, reflexes, and balance. Scores range from 0 to 14, with higher scores indicating more severe neurological impairment.

Motor coordination and sensorimotor function: Motor coordination was assessed using the rotarod test, in which mice were placed on an accelerating rotating rod (4–40 rpm over 5 min), and the latency to fall was recorded. Sensorimotor function was further evaluated using the adhesive removal test and the foot-fault test. In the adhesive removal test, the time required to remove adhesive stickers from the forepaws was recorded (maximum 120 s). In the foot-fault test, the percentage of left forelimb missteps during traversal of a wire grid was calculated. All mice underwent preoperative training to establish stable baseline performance.

Cognitive and social behavioral tests: Social behavior was assessed using the three-chamber sociability test, which measures social preference and social recognition based on interaction time with unfamiliar conspecifics. Cognitive function was evaluated using the novel object recognition test, with recognition index calculated as the proportion of time spent exploring the novel object relative to total object exploration time.

Behavioral assessments were conducted by two independent investigators blinded to group allocation, and inter-rater consistency was confirmed.

### Cerebral blood flow (CBF) measurement

CBF was assessed by laser speckle contrast imaging before pMCAO, and at 4 hours after pMCAO. Mice were anesthetized with isoflurane (3% for induction and 1.5% for maintenance) delivered in oxygen through a nose cone, using the same anesthetic regimen described above, and positioned beneath a charge-coupled device (CCD) camera equipped for laser speckle imaging (RWD Life Science, China). A continuous-wave 785-nm laser was used as the illumination source, and raw speckle images were acquired with an exposure time of 20 ms. Speckle contrast images were generated using the manufacturer’s analysis software. For each animal, five consecutive images from each hemisphere were averaged to reduce noise.

### Euthanasia and tissue collection

At the designated experimental endpoints, mice were deeply anesthetized with sodium pentobarbital (150 mg/kg, intraperitoneally). Adequate anesthetic depth was confirmed by the absence of the pedal withdrawal reflex before terminal procedures. Mice under deep anesthesia were transcardially perfused with phosphate-buffered saline (PBS). Tissues were then rapidly collected. All procedures were performed in accordance with the approved institutional animal protocol (Ethical No. HHYY2025–010) and the AVMA Guidelines for the Euthanasia of Animals. The same euthanasia procedure was used for animals reaching predefined humane endpoints.

### Histological and immunostaining analyses

Brains were harvested 14 d after pMCAO, fixed in 4% paraformaldehyde for 48 hours, dehydrated through graded ethanol, embedded in paraffin, and sectioned coronally at 5 μm thickness. Coronal sections spanning a standardized region encompassing the lesion core (bregma −2.5 mm to +1.0 mm) were prepared for histological and immunostaining analyses.

For lesion volume assessment, seven consecutive coronal sections per brain were stained with hematoxylin and eosin (H&E). Lesion volume was quantified as the percentage of the lesioned cortical area relative to the contralateral hemisphere. All measurements were performed by investigators blinded to experimental group allocation.

For additional histological analyses, Alcian blue–periodic acid–Schiff (AB–PAS) staining was performed to visualize colonic goblet cells according to the manufacturer’s protocol (Solarbio, China).

For immunohistochemistry, paraffin sections were deparaffinized, rehydrated, and subjected to heat-induced antigen retrieval in sodium citrate buffer. After blocking with 5% normal serum, sections were incubated overnight at 4 °C with primary antibodies against GFAP and Iba1. Immunoreactivity was visualized using a DAB-based detection system according to the manufacturer’s instructions. Negative control sections were processed in parallel with omission of the primary antibodies.

For immunofluorescence staining, frozen brain sections or cultured cells were fixed and blocked with serum, followed by overnight incubation at 4 °C with primary antibodies against Iba1, GPR37, and GPR37L1, myelin basic protein (MBP). Sections were then incubated with species-appropriate fluorophore-conjugated secondary antibodies, and lipid droplets were labeled using Bodipy. Nuclei were counterstained with DAPI. Fluorescence images were acquired using a Zeiss LSM 800 confocal microscope under identical acquisition settings. Quantitative analyses of immunoreactivity and fluorescence intensity were performed using ImageJ by investigators blinded to experimental group allocation.

### Western blot analysis

Protein was extracted from frozen tissues or cultured cells using radioimmunoprecipitation assay buffer supplemented with protease and phosphatase inhibitors. Protein concentrations were determined using a bicinchoninic acid assay. Equal amounts of protein were separated by SDS–PAGE and transferred to polyvinylidene fluoride membranes. Membranes were blocked with 5% bovine serum albumin and incubated overnight at 4 °C with primary antibodies against ZO-1, Occludin, PSAP, GPR37, GPR37L1, ERK, *p*-ERK, GAPDH, and *β*-actin. After incubation with horseradish peroxidase–conjugated secondary antibodies, bands were visualized using enhanced chemiluminescence and quantified with ImageJ. GAPDH or *β*-actin served as the loading control.

### Gut microbiota profiling by 16S rDNA sequencing

Genomic DNA was extracted from fecal samples of humans and mice using a commercial kit (AZENTA, USA). The V3–V4 hypervariable regions of the 16S rRNA gene were amplified using universal primers and sequenced on Illumina MiSeq or NovaSeq platforms. After quality filtering and chimera removal, sequences were clustered into amplicon sequence variants and annotated against the SILVA database. Group comparisons were performed using unpaired two-tailed Wilcoxon rank-sum test, with adjusted *P*-values (Padj) for Shannon index, GMHI and MDI, and unadjusted *P*-values for other analyses because only a small number of preselected taxa were analyzed. PCoA was evaluated by Adonis (PERMANOVA) test. Two-sided Spearman’s correlation analysis was used to assess the association between Akk abundance and NIHSS improvement. Receiver operating characteristic (ROC) curve analysis was conducted to evaluate the predictive performance of Akk. All analyses were performed in R (version 4.2.3) with the vegan package on the Majorbio Cloud platform (https://cloud.majorbio.com).

### RNA extraction and quantitative real-time PCR (qRT-PCR)

Total RNA was extracted using TRIzol reagent and reverse-transcribed into complementary DNA. qRT-PCR was performed using SYBR Green chemistry under standard cycling conditions. Relative gene expression was calculated using the ΔΔCt method with *β*-actin as the reference gene. Primer sequences are listed in Table S4.

### ScRNA-seq and analysis

Peri-infarct tissue was collected at 14 d after pMCAO and dissociated into single-cell suspensions with cell viability >80%. Single-cell libraries were generated using the 10x Genomics Chromium Single Cell 3′ v3.1 platform according to the manufacturer’s instructions and sequenced on NovaSeq X Plus or DNBSEQ-T7 platforms.

Raw sequencing data were processed using Cell Ranger (10x Genomics) for read alignment, barcode assignment, and UMI counting. Downstream analyses were performed using Seurat. Cells with fewer than 200 or more than 6000 detected genes, or with >10% mitochondrial gene expression, were excluded. Data were normalized, scaled, and subjected to principal component analysis (PCA), followed by clustering and dimensionality reduction using t-SNE. Batch effects were corrected using the Seurat integration workflow. Cell types were annotated based on canonical marker genes and SingleR reference-based annotation.

Differentially expressed genes were identified using the Wilcoxon rank-sum test with thresholds of |log₂ fold change| > 0.25 and adjusted *P* value (Q value) ≤ 0.05. Gene ontology enrichment analysis was performed using clusterProfiler with multiple testing correction.

### Metabolomic analysis

Mouse plasma samples were subjected to protein precipitation and analyzed using ultra-high-performance liquid chromatography–tandem mass spectrometry (UHPLC–MS/MS) on an Orbitrap Exploris 120 mass spectrometer (Thermo Fisher Scientific) equipped with a BEH Amide column. Data were acquired under both positive and negative ionization modes.

Raw data were converted using ProteoWizard and processed for peak detection, alignment, and normalization (including total ion current normalization) using standard metabolomics workflows implemented in R. Multivariate analyses, including principal component analysis (PCA) and orthogonal partial least squares discriminant analysis (OPLS-DA), were performed to assess global metabolic differences between groups. The validity of the OPLS-DA model was evaluated using permutation testing to avoid overfitting.

Differential metabolites between groups were identified using Student’s t test with Benjamini–Hochberg correction for multiple comparisons. Metabolites with adjusted *P* values (q values) < 0.05 were considered statistically significant. Volcano plots were generated based on log₂ fold change and statistical significance.

Correlation analyses between metabolite levels and behavioral outcomes were performed using Spearman’s correlation. For targeted comparison of key metabolites, including Iso, statistical significance was assessed using Student’s t test.

### Targeted LC-MS/MS for Iso measurement

The levels of Iso in brain tissues and bacterial culture samples were measured by targeted LC-MS/MS. Briefly, samples were extracted with 70% acidified methanol (0.5% formic acid), centrifuged, filtered, and subjected to LC-MS/MS analysis.

Iso quantification was performed using an Agilent 1290 UHPLC system coupled with a 6475 triple quadrupole mass spectrometer. Separation was achieved on a ZORBAX Eclipse Plus C18 column using 0.1% formic acid in water and methanol as mobile phases. Iso was detected in multiple reaction monitoring (MRM) mode using the transition m/z 315.1 −300.1/107.0 and quantified by external calibration with Iso standards.

### Cell culture and *in vitro* ischemia model

BV2 microglial cells and oligodendrocytes were cultured in DMEM supplemented with 10% fetal bovine serum and antibiotics at 37 °C with 5% CO₂. Oxygen–glucose deprivation/reoxygenation (OGD/R) was performed by incubating cells in glucose- and pyruvate-free medium under hypoxic conditions (95% N₂, 5% CO₂), followed by reoxygenation in complete medium. BV2 cells were pretreated with Iso or vehicle as indicated. Conditioned media were collected after reoxygenation and applied to oligodendrocytes. BV2 microglia were pretreated with Iso (50 and 100 μM) for 2 h, then subjected to 4 h of OGD followed by 12 h of reoxygenation. Oligodendrocytes were pretreated with the same concentrations of Iso, PSAP (1 μM), and 50% conditioned medium for 2 h, followed by 8 h of OGD and 12 h of reoxygenation (Supplementary Fig. S7).

### *Psap* and *Gpr37* knockdown

Gene knockdown of *Psap* in BV2 microglia and *Gpr37* in oligodendrocytes was achieved using plasmid-based short hairpin RNA (shRNA) constructs (Jikai Biotechnology, China). Cells were transfected at 50–70% confluence (BV2) or 40–60% confluence (oligodendrocytes) using Lipofectamine 3000 (Thermo Fisher Scientific) according to the manufacturer’s instructions. An empty vector was used as a negative control.

Following transfection, the medium was replaced with fresh complete medium after 4–6 h to minimize cytotoxicity. Knockdown efficiency was validated by qRT–PCR, using FACS-sorted EGFP-positive oligodendrocytes for *Gpr37* and protein analysis (Western blot or enzyme-linked immunosorbent assay, ELISA).

### Cell viability, apoptosis, and ELISA assays

Cell viability was assessed using the Cell Counting Kit-8 assay. Apoptotic cells were detected using terminal deoxynucleotidyl transferase dUTP nick-end labeling (TUNEL) staining, and apoptotic indices were calculated as the percentage of TUNEL-positive nuclei. PSAP levels in BV2 culture supernatants were quantified by ELISA. The above experiments were conducted according to the manufacturer’s instructions (Wuhan Fine Biotech Co., Ltd).

### Adeno-associated virus (AAV)-mediated Gpr37 knockdown and stereotaxic injection

An AAV serotype 9 (AAV9) vector encoding *Gpr37* shRNA (AAV9-Gpr37-RNAi, 3.81 × 10^13^ vg/mL) and corresponding negative control vector (CON688, 2.24 × 10^13^ vg/mL) were obtained from GeneChem Technology (Shanghai, China). A total of 1 μL viral suspension was stereotaxically injected into the right hemisphere targeting the striatum–corpus callosum region to achieve broad diffusion across the striatal white matter region (AP +0.8 mm from bregma, ML +1.6 mm, and DV −3.3 mm from dura). Detailed viral injection parameters and surgical procedures are provided in the Supplementary Materials.

Mice were randomly assigned to six groups:

(1) Ctrl AAV + pMCAO + PBS;
(2) Ctrl AAV + pMCAO + Akk;
(3) Ctrl AAV + pMCAO + Iso;
(4) *Gpr37* knockdown AAV + pMCAO + PBS;
(5) *Gpr37* knockdown AAV + pMCAO + Akk;
(6) *Gpr37* knockdown AAV + pMCAO + Iso.

Drug administration protocols were consistent with those described in the corresponding experimental sections.

### Statistical analysis

Clinical characteristics were summarized using descriptive statistics. Continuous variables are presented as mean ± SD or median (IQR), as appropriate, and categorical variables as counts and percentages. Group comparisons were performed using the χ^2^ test for categorical variables and Student’s t test or Mann–Whitney U test for continuous variables, as appropriate. Because of the limited number of favorable outcome events, Firth’s penalized logistic regression was used for multivariable analyses. The primary model adjusted for age, sex, and dual antithrombotic therapy, followed by one-covariate-at-a-time sensitivity analyses including hypertension, coronary heart disease, atrial fibrillation, diabetes, hyperlipemia, or previous cerebrovascular disease.

For experimental data, values are presented as mean ± SEM unless otherwise indicated. Data normality was assessed using the Shapiro–Wilk test. Comparisons between two groups were performed using two-tailed Student’s t test or Mann–Whitney U test, as appropriate. For multiple group comparisons, one-way or two-way ANOVA followed by appropriate post hoc tests was used. Correlation analyses were performed using Spearman’s correlation, depending on data distribution. Multiple comparisons were corrected using the Benjamini–Hochberg method where appropriate.

All statistical analyses were performed using GraphPad Prism and R.4.6.0. A two-sided *P* value < 0.05 was considered statistically significant. All analyses were performed in a blinded manner where applicable. Sample sizes are indicated in the corresponding figure legends. No data were excluded unless predefined criteria were met.

## Results

### Gut microbiome dysbiosis in IS and its association with early neurological recovery

A total of 86 patients with IS and 28 healthy controls were enrolled in this study according to predefined inclusion and exclusion criteria. Patients with IS exhibited higher rates of hypertension, diabetes mellitus and prior cerebrovascular disease compared with controls, whereas other baseline characteristics were comparable (Supplementary Table S1).

To characterize gut microbiome alterations associated with IS, we performed 16S ribosomal DNA (16S rDNA) sequencing on fecal samples from IS patients and controls (Figure 1A). Compared with controls, IS patients exhibited a significant reduction in microbial *α*-diversity, as identified by a lower Shannon diversity index (Figure 1B), along with a distinct microbial community structure based on *β*-diversity analysis (Figure 1C). Principal coordinates analysis (PCoA) further demonstrated clear separation between IS patients and controls at the genus level (Figure 1D), indicating a global shift in gut microbial composition after stroke. Consistently, the gut microbiome health index (GMHI) was markedly reduced in IS patients (Figure 1E), reflecting an overall deterioration of microbiome-associated health status. Taxonomic analysis revealed substantial compositional remodeling, characterized by depletion of short-chain fatty acid (SCFA)–producing and putatively beneficial genera, including *Lachnospira* and *Butyricicoccus,* and enrichment of potentially pathogenic taxa such as *Escherichia–Shigella* (Figure 1F, G). Together, these findings indicate that IS is associated with reduced microbiome diversity and a dysbiotic gut ecosystem.

To determine whether gut microbiome composition is associated with early neurological recovery, IS patients were further stratified into a favorable outcome group and the other-outcome group based on changes in NIHSS score between admission and discharge (Figure 1H). Baseline clinical characteristics were comparable between groups (Supplementary Table S2). *β*-diversity analysis revealed a significant difference in overall microbial community structure between outcome groups (Figure 1I), indicating that gut microbiome composition varies with early recovery. Consistently, the microbial dysbiosis index (MDI) was significantly lower in patients in the favorable outcome group (Figure 1J), suggesting reduced microbiome imbalance in patients with better recovery. Genus-level analysis identified distinct microbial signatures associated with clinical outcomes (Figure 1K). Notably, *Akkermansia muciniphila* was among the most significantly enriched taxa in patients with a favorable recovery and its relative abundance was higher in this group compared with the other-outcome group (Figure 1L). Furthermore, Akk abundance showed a positive correlation with improvement in NIHSS score (*r* = 0.2637, *p* = 0.0142; Figure 1M). Receiver operating characteristic (ROC) analysis yielded an area under the curve (AUC) of 0.91 (95% confidence interval [CI]: 0.85–0.97), indicating that microbiome-derived features, including Akk abundance, are associated with early neurological outcomes (Figure 1N). Together, these results demonstrate that early IS is associated with gut microbiome dysbiosis and identify Akk as a microbiota-associated taxon linked to early neurological recovery.

To account for potential clinical confounders, multivariable Firth penalized logistic regression was performed. Higher Akk abundance remained significantly associated with favorable early neurological recovery after adjustment for age, sex, and dual antithrombotic therapy (adjusted odds ratio (OR) = 1.97, 95% CI: 1.20–3.35, *p* = 0.008). The association remained robust in sensitivity analyses (Supplementary Table S3).

**Figure 1.**
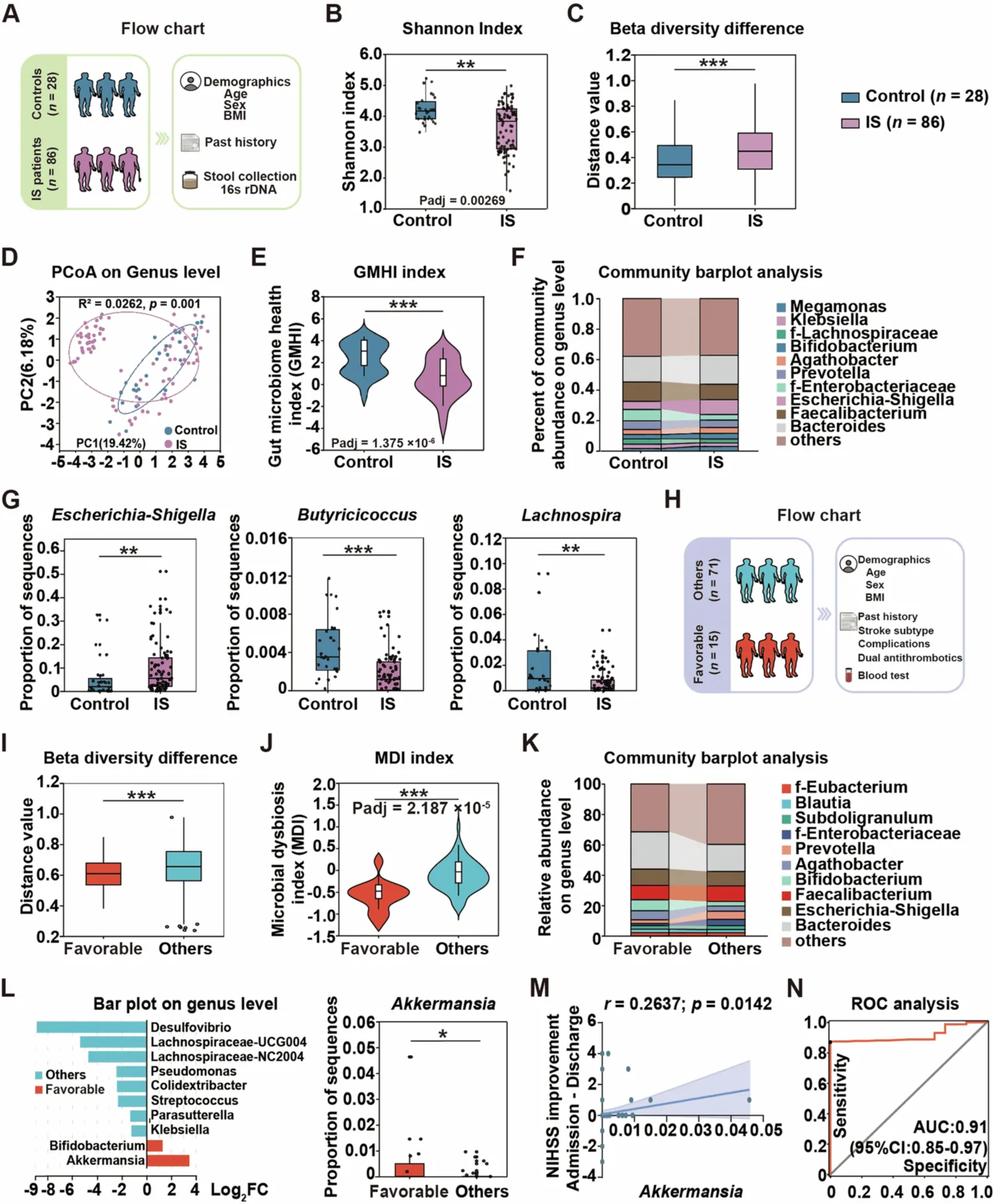
Microbial diversity analysis with 16S rDNA. (A) Study participants inclusion, group division and sample collection. (B) *α*-diversity and (C) *β*-diversity analysis between control and IS groups. (D) PCoA diagram on the genus level analyzed the differences in community composition. (E) Comparison of the GMHI between control and IS groups. (F) Microbial composition based on relative abundance between control and IS groups at the genus level. (G) Analysis of the relative abundance of different bacterial at the genus level. (H) Group division and data collection of IS patients. (I) *β*-diversity analysis between favorable prognosis group and other-outcome group. (J) Comparison of the MDI between favorable prognosis group and an other-outcome group. (K) Microbial composition based on relative abundance between favorable prognosis group and an other-outcome group at the genus level. (L) The enriched differential microbiota in different groups on genus level with bar plot and analysis of the Akk relative abundance at the genus level. (M) Spearman correlation analysis between the Akk and prognosis of IS. (N) ROC analysis of Akk for distinguishing favorable outcome group from others. (B, C, E, G, I, J, L): Unpaired two-tailed Wilcoxon rank-sum test. (D): Adonis (PERMANOVA) test. (M): Spearman's rank correlation coefficient. (N): Receiver operating characteristic (ROC) curve analysis. *p < 0.05, **p < 0.01, ***p < 0.001. Data in (B, C, E, G, I, J, L) are presented as mean ± SD. Each dot represents a patient.

### Akk supplementation promotes functional recovery after IS independent of infarct size

To determine whether Akk directly influences post-stroke recovery, mice subjected to pMCAO followed by treatment with PBS, low-dose Akk, or high-dose Akk, and performed longitudinal behavioral and histological assessment (Figure 2A). Laser speckle imaging confirmed a marked reduction in cortical CBF after pMCAO, validating successful model establishment (Figure 2B).

Behavioral analyses revealed a dose-dependent effects of Akk on neurological recovery. Compared with PBS-treated mice, low-dose Akk treatment produced modest improvements, reaching statistical significance only in the rotarod test at later time points. In contrast, high-dose Akk treatment resulted in robust and sustained improvements across multiple neurological domains during the recovery phase. Specifically, high-dose Akk significantly reduced mNSS, decreased forelimb foot-fault rates, shortened adhesive removal latency, and improved motor coordination on the rotarod, with effects emerging as early as days 3–7 after stroke (Figure 2C). Direct comparison further confirmed superior efficacy of high-dose Akk over low-dose Akk in multiple behavioral outcomes, supporting a dose–response relationship.

We next assessed cognitive function using the novel object recognition test and the three-chamber social interaction test. PBS-treated mice exhibited impaired recognition memory compared with sham controls, whereas high-dose Akk treatment significantly improved the recognition index (Figure 2D). Consistently, while overall sociability was not significantly altered, social novelty preference was impaired after stroke and selectively restored by high-dose Akk treatment (Figure 2E).

To determine whether Akk affects acute injury severity, we evaluated infarct-related brain atrophy at 14 d after stroke. No significant differences were observed between PBS-, and high-dose Akk–treated mice (Figure 2F). These findings indicate that Akk improves neurological and cognitive recovery without significantly altering infarct size, suggesting that its beneficial effects are unlikely to be mediated primarily through acute neuroprotection.

Given the known role of Akk in regulating intestinal homeostasis, we next examined whether Akk treatment affected stroke-induced gut alterations. Akk partially alleviated intestinal inflammation and microbiota dysbiosis without significantly altering its own abundance (Supplementary Figs. S1 and S2), suggesting that its beneficial effects are unlikely to result from simple bacterial expansion.

Although Akk treatment altered specific bacterial taxa, including restoration of *Turicibacter* abundance, administration of *Turicibacter* alone failed to recapitulate the protective effects (Supplementary Fig. S3), indicating that Akk-mediated benefits are unlikely to be driven by individual microbial taxa but instead may depend on downstream metabolic mechanisms, prompting further investigation into Akk-derived metabolites.

**Figure 2.**
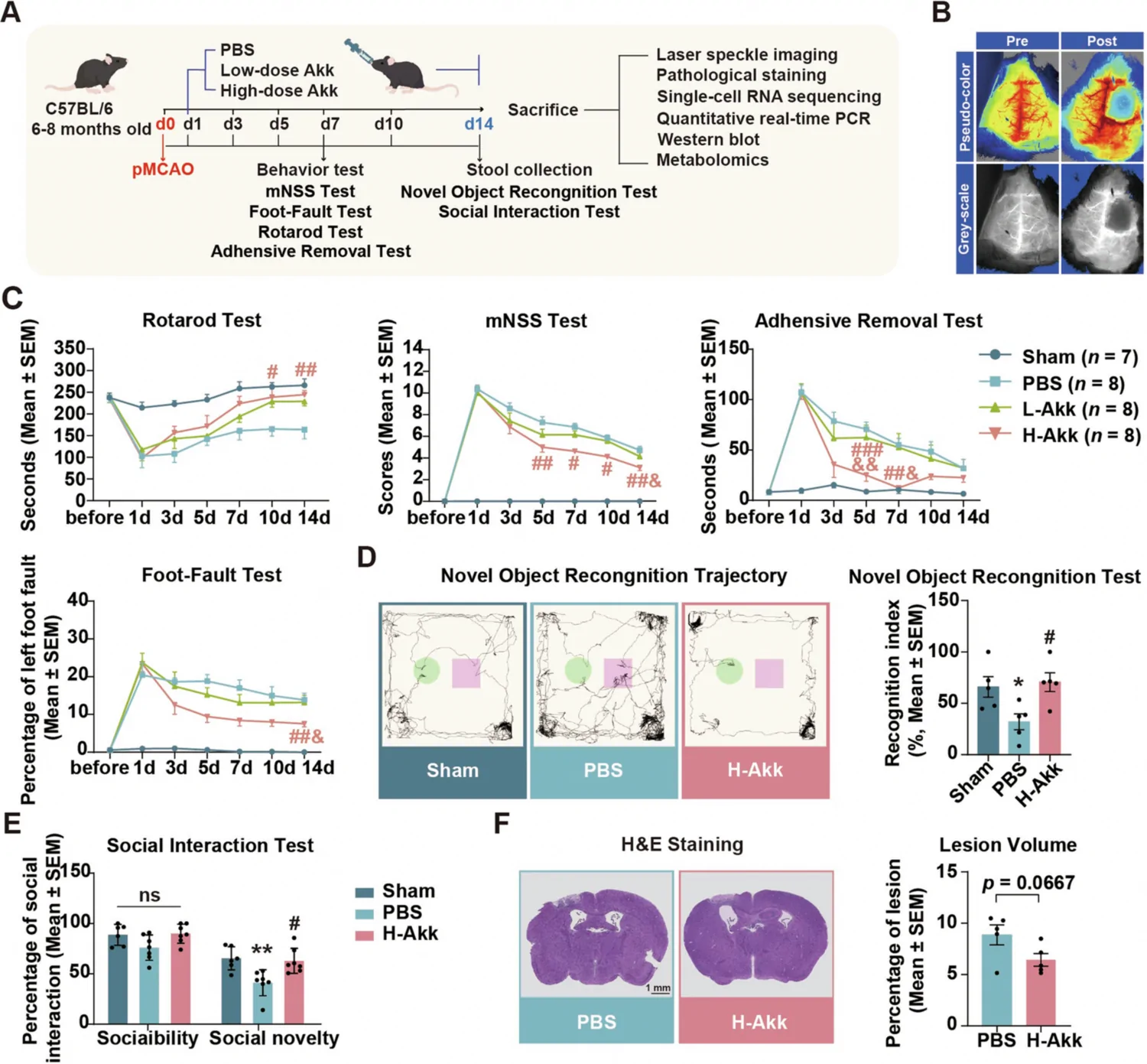
Effects of Akk on pMCAO model with behavioral tests and brain lesion assessment. (A) Schematic illustration of the experimental design. 6-8-month-old C57BL/6 mice were subjected to pMCAO. Mice were administered intragastrically with PBS, low-dose Akk (L-Akk) or high-dose Akk (H-Akk) from day 0 to day 14. A series of behavioral tests were performed, and samples were collected at day 14 for further analyses. (B) Laser speckle imaging present perfusion maps before and after pMCAO. (C) Neurological function assessments including rotarod test, mNSS test, adhesive removal test and foot-fault test at different time points after stroke. (D) Novel object recognition test and (E) social interaction test among Sham, PBS and H-Akk groups. (F) H&E-stained brain sections and quantification of brain lesion volume in PBS and H-Akk groups. (C): Intergroup differences at each time point were analyzed using One-way ANOVA, followed by Holm modified Šidák post-hoc test; for normally distributed data and Kruskal-Wallis H test, followed by Dunn’s post-hoc test for non-normally distributed data. (D): One-way ANOVA, followed by Holm modified Šidák post-hoc test for multiple comparisons. (E): Kruskal-Wallis H test, followed by Dunn’s post-hoc test for multiple comparisons. (F): Student’s t test. *p < 0.05, **p < 0.01 vs Sham group; ^#^ *p* < 0.05 vs PBS group; ^&^ *p* < 0.05 vs L-Akk group; ns, not significant. Each dot indicates an individual mouse.

### Akk suppresses lipid-associated microglia activation and promotes white matter repair after ischemic stroke

To investigate the cellular mechanisms underlying Akk-mediated post-stroke recovery, we performed histological and molecular analyses in the peri-infarct brain at 14 d after pMCAO (Figure 3). Immunohistochemical staining for Iba1 revealed robust microglial activation in PBS-treated mice, whereas Akk-treated mice exhibited a significant reduction in Iba1-positive area. Morphological assessment using Sholl analysis further showed that Akk treatment preserved a more ramified microglial morphology, indicative of a homeostatic state (Figure 3A).

Given the emerging role of lipid metabolic reprogramming in shaping microglial activation after brain injury, we next assessed lipid droplet accumulation using Bodipy staining. In PBS-treated mice, lipid droplets were markedly increased and extensively colocalized with Iba1-positive microglia, consistent with the formation of lipid-laden “foamy” microglia. Akk treatment substantially reduced microglia-associated lipid droplet accumulation (Figure 3B), suggesting suppression of this maladaptive microglial state. Consistent with these observations, qRT-PCR revealed that pMCAO markedly increased the expression of pro-inflammatory cytokines, including *Tnfα*, and *Il6*, compared with sham-control mice. Akk treatment significantly attenuated this response (Figure 3C), indicating reduced neuroinflammation. We next assessed whether these cellular changes were associated with white matter integrity. Immunofluorescence analysis of MBP revealed a significant reduction in MBP-positive area in PBS-treated mice, whereas Akk-treated mice showed increased MBP signal in the peri-infarct region, including the corpus callosum and striatum (Figure 3D). Together, these findings indicate that Akk suppresses lipid-associated, pro-inflammatory microglial activity and is associated with improved white matter integrity after ischemic stroke.

**Figure 3.**
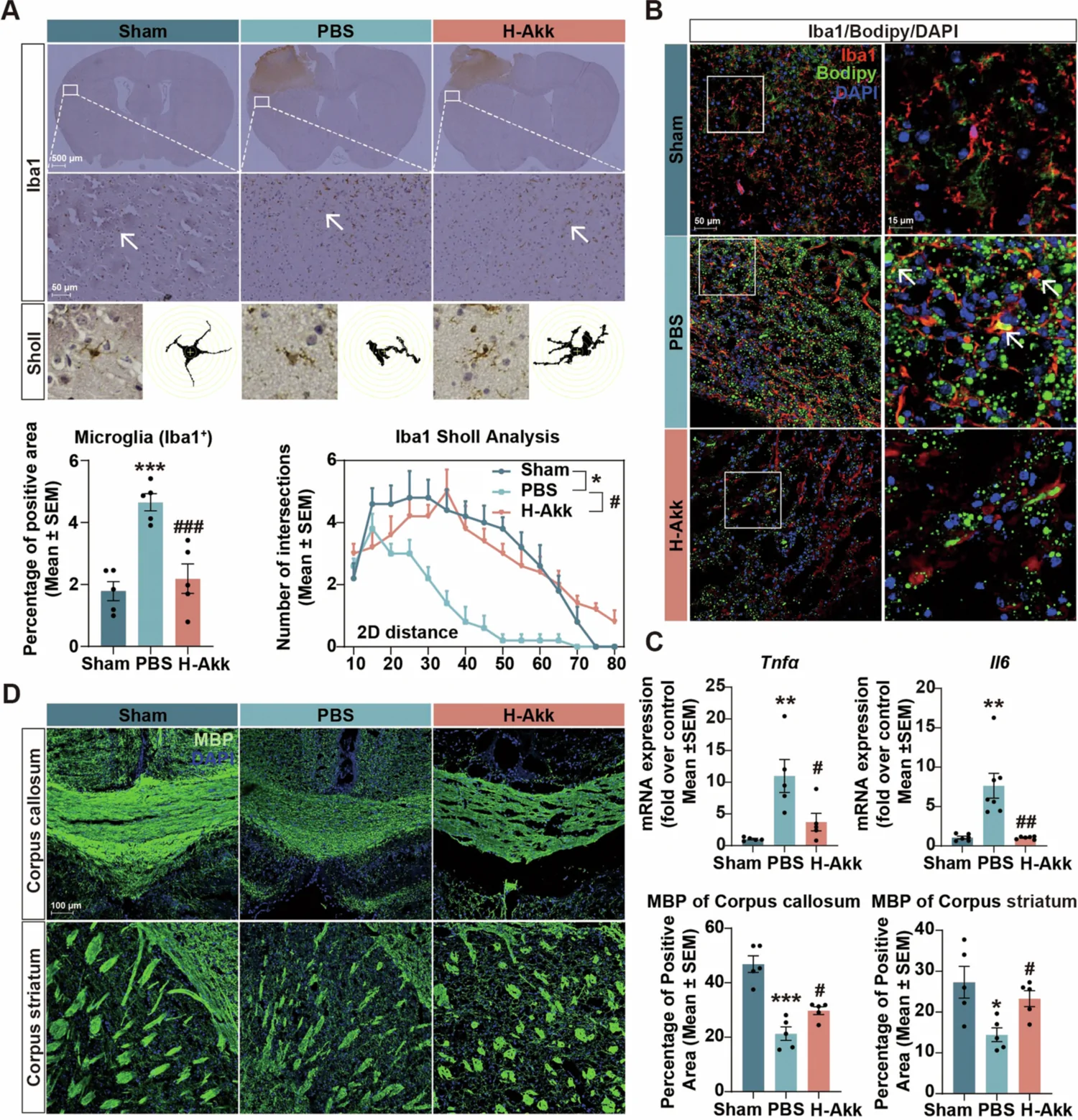
Results of pMCAO mouse model with Akk treatment on neuroinflammation, microglial lipid droplet accumulation, and white matter injury. (A) Representative IHC staining for Iba1 (microglia marker) of cortical regions surrounding the cerebral infarction in Sham, PBS, and H-Akk groups, with quantification of the Iba1-positive area and Sholl analysis of microglial morphology. (B) Representative Immunofluorescence staining for Bodipy (lipid droplets marker), Iba1 and DAPI (nuclei marker). (C) qRT-PCR analysis of pro-inflammatory cytokine mRNA expression in the ischemic boundary zone brain at 14 d post-pMCAO. (D) Representative immunofluorescence staining for MBP (myelin basic protein marker) and DAPI, and quantitative analysis of MBP percentage area in the corpus callosum and corpus striatum. (A): One-way ANOVA for microglia analysis, followed by Holm modified Šidák post-hoc test for multiple comparisons, and Kruskal-Wallis H test for Sholl analysis was performed at each distance to assess intergroup differences, followed by Dunn’s post-hoc test for pairwise comparisons. (C): One-way ANOVA for TNF-*α* analysis, followed by Dunnett’s post-hoc test for multiple comparisons; Kruskal-Wallis H test for IL-6 analysis, followed by Dunn’s post-hoc test for multiple comparisons. (D): One-way ANOVA, followed by Holm modified Šidák test for multiple comparisons. **p < 0.01, ***p < 0.001 vs Sham group; ^#^ *p* < 0.05, ^##^ *p* < 0.01, ^###^ *p* < 0.001 vs PBS group. Data in (A, C, D) are presented as mean ± SEM. Each dot indicates an individual mouse.

### Single-cell RNA sequencing reveals Akk-induced microglial reprogramming and enhanced oligodendrocyte maturation through PSAP–GPR37-mediated communication after ischemic stroke

To further define the cellular mechanisms underlying Akk-mediated white matter repair, we performed single-cell RNA sequencing (scRNA-seq) of peri-infarct cortical tissue from sham, PBS-treated, and high-dose Akk–treated mice at 14 d after pMCAO (Figure 4). After quality control and unsupervised clustering, cells were classified into 14 major cell types based on canonical marker gene expression, including microglia, oligodendrocytes, astrocytes, endothelial cells, and oligodendrocyte precursor cells (OPCs). Comparative analysis revealed a marked expansion of microglia in PBS-treated mice, which was significantly attenuated by Akk treatment (Figure 4A), indicating that microglia represent a major cellular response to Akk in the post-stroke brain.

To further resolve microglial heterogeneity, we identified four transcriptionally distinct microglial states (Figure 4B). Homeostatic microglia, characterized by expression of *Tmem119*, *P2ry12*, *Sall1*, and *Cx3cr1*, predominated in sham brains but were markedly reduced after stroke and was partially restored by Akk treatment. In contrast, stroke induced a pronounced expansion of lipid-associated, foamy microglia, enriched for genes involved in lipid metabolism and phagocytic, including *Plin2*, *Apoe*, *Trem2*, *Lpl*, *Cd36*, and *Cst7.* This population was markedly reduced following Akk treatment. Akk also promoted the emergence of axon tract–associated microglia (ATM), expressing *Clec7a*, *Fabp5*, *Spp1*, and *Gpnmb*, while reducing disease-associated microglia (DAM) linked to chronic inflammation and neurodegeneration (Supplementary Fig. S4A).

To determine how Akk-induced microglial reprogramming influences intercellular communication, we performed ligand–receptor interaction analysis. Microglia exhibited extensive signaling interactions with multiple neural cell types, with particularly strong connectivity to oligodendrocytes and OPCs (Supplementary Fig. S4B). Among the top-ranked signaling pathways, the PSAP–Gpr37 axis emerged as a prominent microglia-to-oligodendrocyte communication route (Figure 4C). Network centrality analysis further identified microglia as key signal senders within the PSAP signaling network, whereas oligodendrocytes primarily functioned as signal receivers (Figure 4D).

Together, these data indicated that Akk treatment reprograms microglial states away from lipid-associated, pro-inflammatory phenotypes and enhances microglia–oligodendrocyte communication through the PSAP–GPR37 pathway, supporting a potential mechanism linking microbiota-derived signals to white matter repair after IS.

To determine whether Akk-mediated microglial reprogramming is associated with changes in oligodendrocyte lineage dynamics, we re-clustered oligodendrocytes from the single-cell dataset and identified six transcriptionally distinct subclusters. Quantitative analysis revealed shifts in subcluster composition across experimental groups, with high-dose Akk treatment significantly increasing the proportion of a subcluster enriched for myelination-associated gene expression compared with PBS-treated mice (Figure 4E). This subcluster was characterized by high expression of canonical myelination-associated genes, including *Mag*, *Mog*, *Mbp*, and *Plp1*, consistent with a mature oligodendrocyte transcriptional profile (Supplementary Fig. S4C). Differential gene expression analysis followed by Gene Ontology enrichment further showed that genes upregulated in Akk-treated mice were associated with pathways related to myelination, compact myelin formation, and lipid binding (Figure 4F), indicating enhanced activation of myelination programs.

To further assess oligodendrocyte precursor cells (OPC) progression, we performed pseudotime trajectory analysis. In PBS-treated mice, OPC were predominantly positioned at early pseudotime states, suggesting impaired progression along the differentiation trajectory after ischemic injury. In contrast, cells from high-dose Akk–treated mice were preferentially enriched at later pseudotime states, indicative of accelerated progression toward mature, myelinating oligodendrocytes (Figure 4G), consistent with enhanced progression toward myelinating oligodendrocyte states.

Together, these findings suggest that Akk treatment is associated with a shift in oligodendrocyte lineage dynamics toward myelination-associated states, providing a potential cellular link between microglial reprogramming and white matter repair after ischemic stroke.

**Figure 4.**
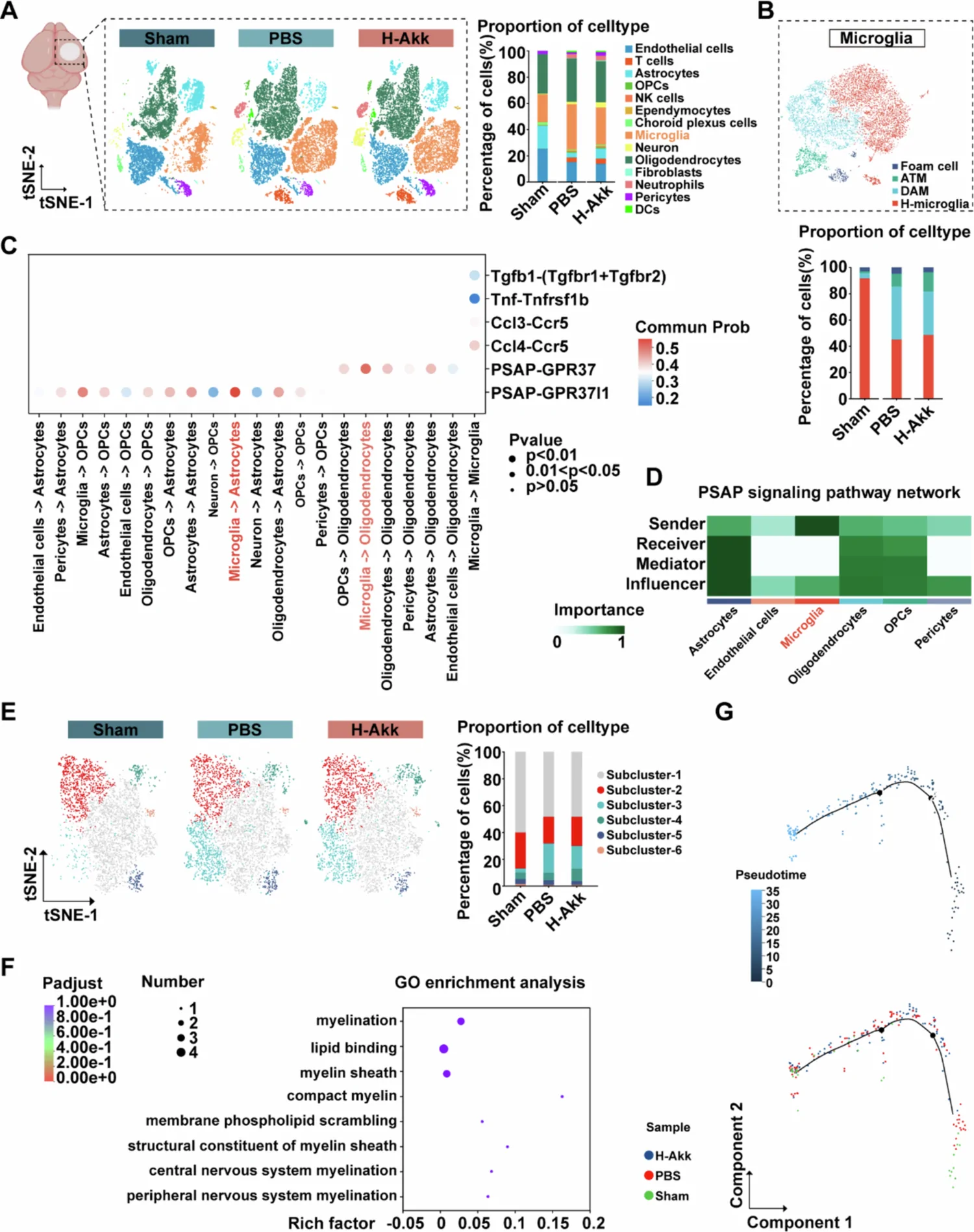
ScRNA-seq of the surrounding tissues of the IS area. (A) The tSNE-1 of 14 distinct cell types identified by unsupervised clustering. (B) The t-Distributed Stochastic Neighbor Embedding (t-SNE)-1 of microglia showing distinct microglial subpopulations: Foam cell, ATM, DAM, H-microglia. (C) Communication probability of key ligand-receptor across different cell type pairs, with significance indicated by *p* values. (D) PSAP signaling pathway network analysis, showing the importance of “Sender-Receiver-Mediator-Influencer” across Microglia and other cell types. (E) tSNE plots of single cells from Sham, PBS, and H-Akk groups, identified six subclusters. Stacked bar chart showing the proportion of each subcluster across groups. (F) GO enrichment analysis highlighting myelination-related pathways enriched in H-Akk group. (G) Pseudotime trajectory analysis: cells ordered by pseudotime and by experimental group.

### Akk activates oligodendrocyte-associated PSAP–GPR37–ERK signaling after IS

To determine whether the PSAP–GPR37 pathway identified by scRNA-seq analysis is engaged following Akk treatment, we performed molecular and histological validation in peri-infarct brain tissue. qRT-PCR analysis revealed that *Psap* and *Gpr37* mRNA levels were significantly reduced in PBS-treated ischemic mice compared with sham controls, whereas high-dose Akk administration restored their expression (Figure 5A).

Consistently, immunoblot analysis demonstrated increased PSAP and GPR37 protein levels in Akk-treated mice, accompanied by enhanced phosphorylation of ERK, a downstream effector of GPR37 signaling cascade (Figure 5B).

To assess pathway specificity, we examined GPR37L1, an alternative PSAP receptor predominantly expressed in astrocytes. Neither mRNA nor protein levels of GPR37L1 were altered by Akk treatment (Supplementary Fig. S5), supporting selective engagement of the PSAP–GPR37 axis.

Immunofluorescence analysis further showed that GPR37 expression was increased in the peri-infarct region after Akk treatment and was predominantly localized to Olig2^+^ oligodendrocyte lineage cells, indicating that oligodendrocytes represent a principal cellular target of PSAP–GPR37 signaling *in vivo* (Figure 5C). Together, these results indicate that Akk administration is associated with activation of a PSAP–GPR37–ERK signaling axis in oligodendrocyte lineage cells, supporting a mechanistic link between Akk-induced microglia reprogramming and oligodendrocyte-associated repair response after IS.

**Figure 5.**
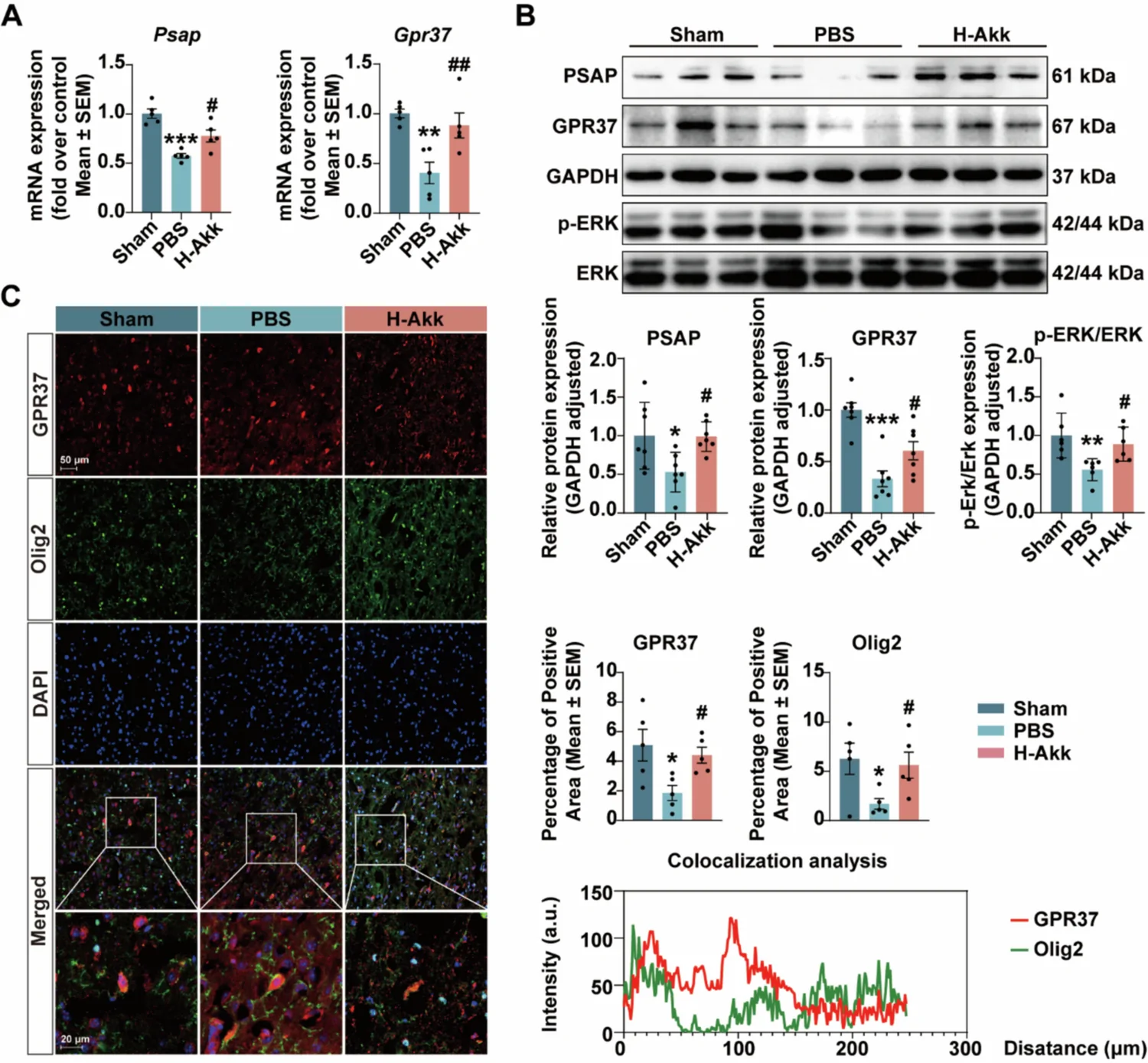
Akk modulates PSAP/GPR37 expression and ERK phosphorylation. (A) qRT-PCR analysis of *Psap* and *Gpr37* mRNA expression. (B) Representative Western blots and quantitative analysis of PSAP, GPR37, *p*-ERK/ERK protein levels. (C) Representative immunofluorescence staining of GPR37, Olig2, and DAPI, quantification of GPR37- and Olig2-positive areas, and line profiles of fluorescence intensity. (A): One-way ANOVA, followed by Holm modified Šidák post-hoc test for multiple comparisons. (B): One-way ANOVA for PSAP and GPR37 analysis, followed by Holm modified Šidák post-hoc test for multiple comparisons; Kruskal-Wallis H test for *p*-ERK/ERK analysis, followed by Dunn’s post-hoc test for multiple comparisons. (C): One-way ANOVA, followed by Holm modified Šidák post-hoc test for multiple comparisons. *p < 0.05, **p < 0.01 ***p < 0.001 vs Sham group; ^#^ *p* < 0.05, ^##^ *p* < 0.01 vs PBS group. Data in (A–C) are presented as mean ± SEM. Each dot indicates an individual mouse.

### Akk supplementation increases circulating Iso, which correlates with neurological recovery after IS

To identify systemic mediators linking Akk supplementation to brain repair, we performed untargeted plasma metabolomic profiling in sham, PBS-treated, and high-dose Akk–treated mice at 14 d after pMCAO. Principal component analysis (PCA) revealed clear separation among groups, indicating Akk-dependent reprogramming of systemic metabolism (Figure 6A). Comparative analysis identified 372 differentially abundant metabolites between PBS-treated and sham mice and 398 metabolites between high-dose Akk and PBS-treated mice (Figure 6B). Among the metabolites altered by Akk supplementation, 69 were significantly increased. Notably, volcano plot analysis identified Iso as one of the most prominently upregulated metabolites in the Akk-treated mice (Figure 6C), which was further confirmed by quantitative abundance analysis (Figure 6D).

Importantly, circulating Iso levels were strongly associated with functional outcomes. Iso abundance was negatively correlated with neurological deficit severity (mNSS and foot-fault) and positively correlated with motor coordination (rotarod performance) (Figure 6E), identifying Iso as a candidate systemic mediator of Akk-associated neurological recovery.

Pathway classification of Akk-regulated metabolites revealed a predominance of lipid-related pathways, with phospholipids representing the most enriched metabolite class (Figure 6F). Among these, multiple sphingomyelins (SM) species (key structural components of myelin sheaths)^18^ were significantly increased following Akk treatment (Figure 6G). Importantly, the abundance of these sphingomyelins positively correlated with circulating Iso levels (Figure 6H), and several species were also associated with improved neurological outcomes (Supplementary Fig. S6), suggesting coordinated regulation of lipid metabolism in parallel with Iso elevation.

Together, these data identify Iso as a prominent Akk-induced circulating metabolite associated with improved neurological recovery and lipid metabolic remodeling after IS, supporting a potential role for metabolite-mediated signaling in Akk-driven brain repair.

**Figure 6.**
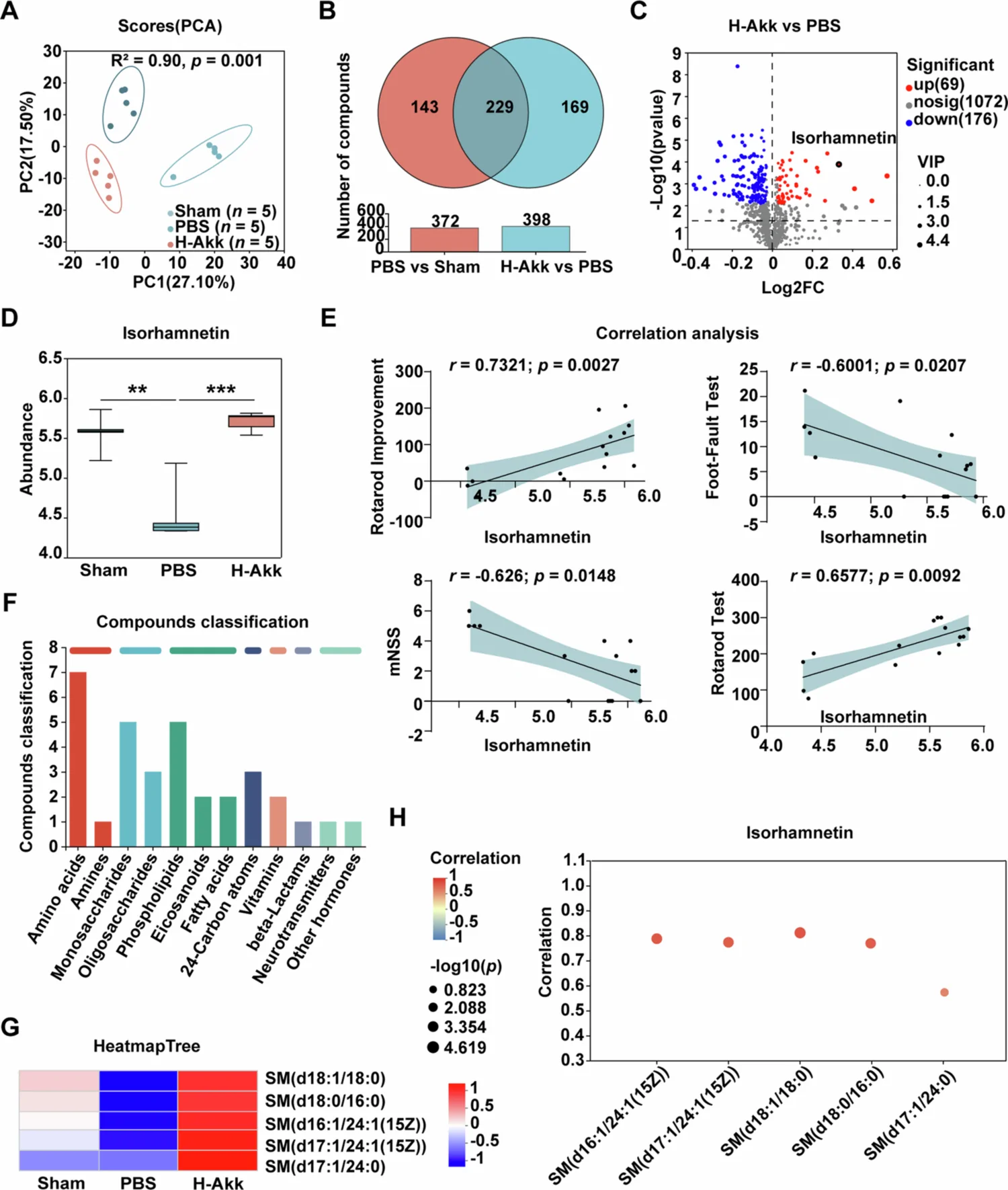
Metabolomic analyses reveal Iso and sphingomyelin alterations associated with Akk-mediated post-stroke recovery. (A) PCA plot shows distinct metabolic profiles among Sham, PBS, and H-Akk groups. (B) Venn diagram and bar plot illustrating the number of differential metabolites between Sham vs PBS and PBS vs H-Akk. (C) Volcano plot of differential metabolites in H-Akk vs PBS, highlighting Iso. (D) Abundance of isorhamnetin across Sham, PBS, and H-Akk groups. (E) Correlation analyses between isorhamnetin levels and behavioral outcomes (mNSS, Foot-fault test, Rotarod improvement, Rotarod test). (F) Classification of differential compounds, with phospholipids being the main abundant category. (G) HeatmapTree depicting the expression patterns of sphingomyelins (SMs) across groups. (H) Correlation plot showing positive associations between isorhamnetin and specific SM species. (A): Adonis (PERMANOVA) test. (B–D): Unpaired student’s t test, *p* value (B) and Padj (C, D) were used. (E): Spearman correlation analysis. (H) Pearson’s correlation analysis. **p < 0.01, ***p < 0.001. Data in (D) are presented as mean ± SD. Each dot indicates an individual mouse.

### Iso phenocopies Akk-mediated functional recovery and enhances the PSAP–GPR37–ERK axis to suppress microglial lipid accumulation and promote white matter repair

To test whether the Akk-induced increase in circulating Iso is sufficient to mediate post-stroke recovery, pMCAO mice were treated with Iso or PSAP administration included as a pathway-relevant comparator (Figure 7A). Longitudinal behavioral assessments revealed that Iso administration significantly improved neurological recovery, as evidenced by reduced mNSS scores and improved performance in the foot-fault and adhesive removal tests compared with PBS-treated mice (Figure 7B). At 14 d after pMCAO, Iso-treated mice also exhibited marked improvements in social novelty in the three-chamber social interaction test (Figure 7C). Notably, PSAP treatment produced comparable improvements across neurological and cognitive outcomes, suggesting convergence on a shared downstream repair pathway. Together, these findings indicate that Iso is sufficient to recapitulate the functional benefits of Akk supplementation after IS.

To determine whether Akk-associated Iso elevation contributes to activation of the PSAP–GPR37 signaling pathway, we investigated the effect of Iso supplementation on this downstream repair axis identified by scRNA-seq. Targeted LC-MS/MS analysis demonstrated that H-Akk treatment significantly increased Iso abundance in brain tissue compared with PBS-treated mice, whereas Iso levels were comparable between Akk-conditioned medium and control medium, suggesting that Akk treatment is associated with increased cerebral Iso availability rather than direct Iso production by Akk, thereby supporting its potential contribution to PSAP–GPR37 signaling activation (Figure 7D). Moreover, both Iso and PSAP treatment significantly increased PSAP and GPR37 protein levels and enhanced ERK phosphorylation in peri-infarct brain tissue (Figure 7E), indicating activation of this signaling cascade. Functionally, Iso treatment markedly reduced lipid droplet accumulation in Iba1⁺ microglia (Figure 7F). consistent with suppression of lipid-associated microglial activation. In parallel, immunofluorescence staining demonstrated that Iso and PSAP restored MBP expression in the corpus callosum and striatum following pMCAO (Figure 7G), indicating improved white matter integrity.

Together, these findings suggest that Akk-associated elevation of Iso contributes to activation of the PSAP–GPR37–ERK signaling pathway and promotes microglia–oligodendrocyte-mediated repair after ischemic stroke. These data identify Iso as a potential metabolic mediator linking microbiota-associated metabolic remodeling with endogenous brain repair mechanisms.

**Figure 7.**
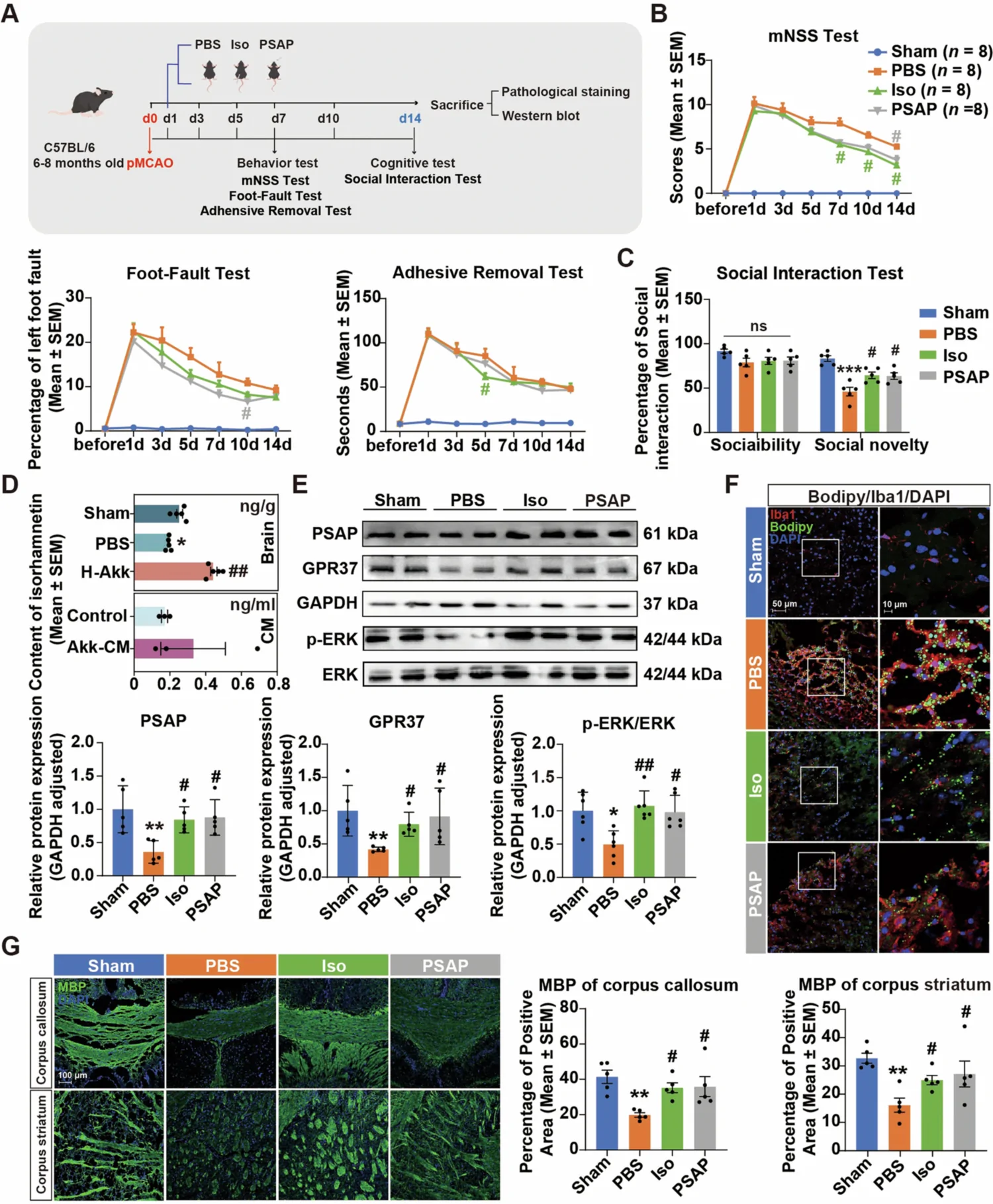
Effects of Iso on neurological function, PSAP/GPR37/ERK signaling, lipid deposition and myelin repair after pMCAO. (A) Schematic timeline of the experimental protocol: 6–8-month-old C57BL/6 mice underwent pMCAO, followed by PBS, intraperitoneal injection of Iso, or nasal administration of PSAP on day 1 post-stroke. Behavioral tests were performed at the indicated time points, and mice were euthanized at day 14 using the procedure described. (B) Behavioral outcomes post-stroke: mNSS, foot-fault test, adhesive removal test. (C) Cognitive assessments: Social Interaction Test. (D) Targeted LC-MS/MS analysis of isorhamnetin levels in brain tissues from Sham, PBS, and H-Akk-treated mice, and in control medium and Akk-conditioned medium (CM). (E) Representative Western blots of PSAP, GPR37, *p*-ERK/ERK. (F) Immunofluorescence staining of Bodipy, Iba1 and DAPI. (G) Immunofluorescence staining of MBP in corpus callosum and corpus striatum. (B): Intergroup differences at each time point were analyzed using One-way ANOVA, followed by Holm modified Šidák post-hoc test; for normally distributed data and Kruskal-Wallis H test, followed by Dunn’s post-hoc test for non-normally distributed data. (C, G): One-way ANOVA, followed by Holm modified Šidák post-hoc test. (E): One-way ANOVA for PSAP and *p*-ERK/ERK analysis, followed by Holm modified Šidák post-hoc test, Kruskal-Wallis H test for GPR37 analysis, followed by Dunn’s post-hoc test for multiple comparisons. (D) Brain tissue Iso concentrations were analyzed using one-way ANOVA followed by Holm–Šidák’s multiple-comparisons test. Iso concentrations in the culture medium were compared between the two groups using a two-tailed Student’s t-test. *p < 0.05, **p < 0.01 vs Sham group; ^#^ *p* < 0.05, ^##^ *p* < 0.01, ^###^ *p* < 0.001 vs PBS group; ns, not significant. Data in (B, C, E, G) are presented as mean ± SEM. Each dot indicates an individual mouse.

### Iso–PSAP–GPR37 signaling mediates microglia–oligodendrocyte crosstalk to promote oligodendrocyte survival under ischemic stress

To directly determine whether Iso regulates microglia–oligodendrocyte communication through PSAP–GPR37 signaling, we performed complementary loss-of-function and conditioned-medium experiments.

First, BV2 microglia subjected to OGD/R exhibited robust inflammatory activation and lipid droplet accumulation, both of which were markedly attenuated by Iso treatment (Figure 8A, B, D). Iso significantly increased *Psap* mRNA expression and secretion, as confirmed by qRT–PCR and ELISA (Figure 8C). Importantly, knockdown of *Psap* in microglia abolished the anti-inflammatory effects of Iso, reduced PSAP secretion, and reversed its lipid-lowering effects (Figure 8E–H), demonstrating that PSAP is required for Iso-mediated microglial reprogramming.

**Figure 8.**
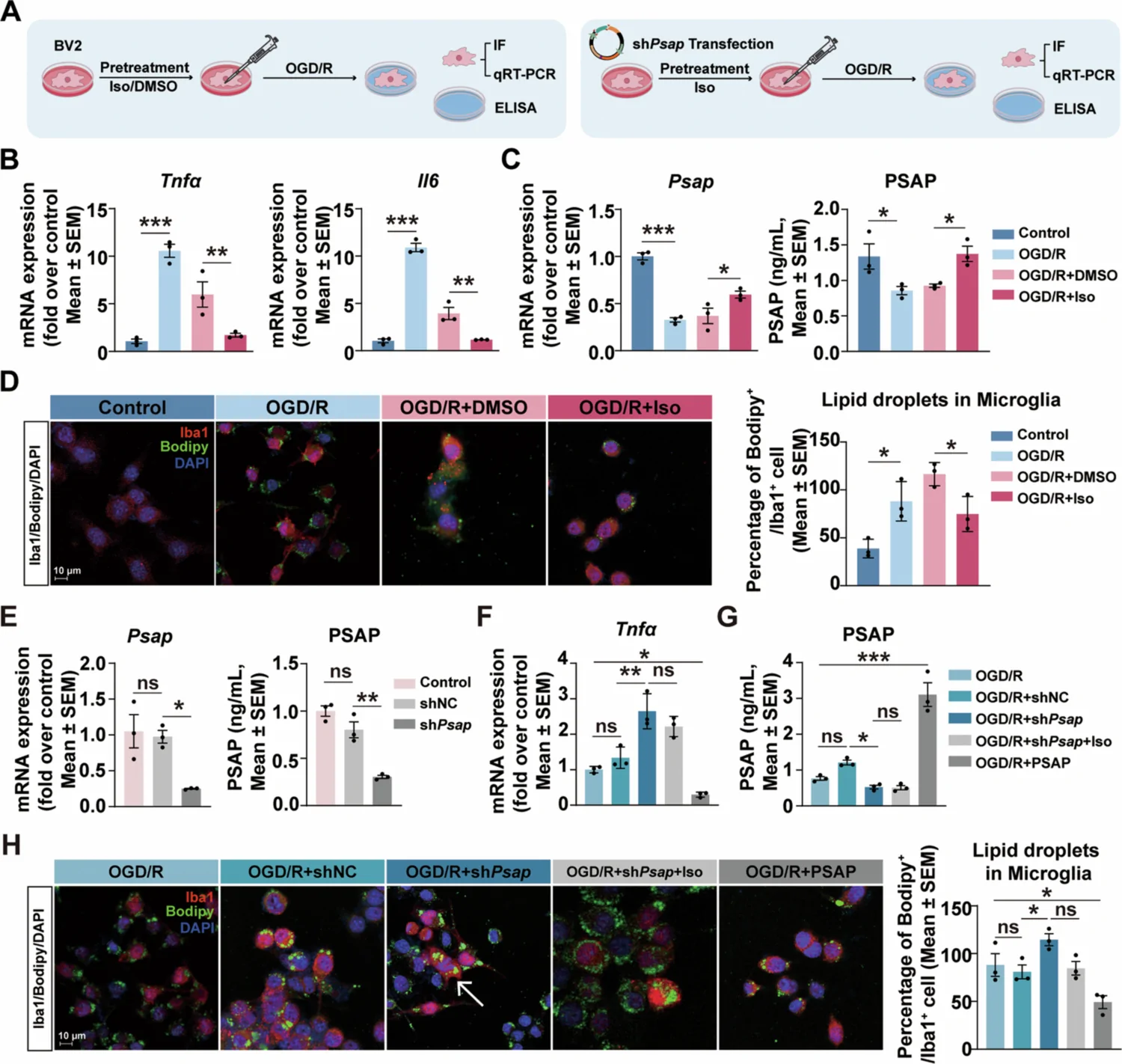
Iso modulates microglial responses to OGD/R via PSAP-dependent signaling *in vitro*. (A) Schematic overview of the experimental design. (B) qRT–PCR analysis of inflammatory markers (*Tnfα* Il6) expression in BV2 microglia. (C) qRT-PCR analysis of *Psap* mRNA and ELISA quantification of PSAP secretion in microglial supernatants. (D) Iba1, BODIPY, and DAPI staining and quantification of lipid droplet accumulation. O GD/R increased lipid droplet accumulation, which was reduced by Iso treatment. (E) Reduced Psap mRNA expressi on and *Psap* secretion following *Psap* knockdown. (F–H) Effects of *Psap* knockdown on Iso-mediated regulation of (F) increased Tnf-α mRNA expression following *Psap* knockdown, with no significant change after Iso treatment in *Psap*-knockdown cells, (G) Reduced *PSAP* secretion following *Psap* knockdown, with no significant change after Iso treatment in *Psap*-knockdown cells, and (H) Immunofluorescence images and quantification of lipid droplet accumulation. *Psap* knockdown increased lipid droplet accumulation, which was not sig nificantly altered by Iso treatment.. (B–H): One-way ANOVA, followed by Holm modified Šidák post-hoc test. *p < 0.05, **p < 0.01, ***p < 0.001; ns, not significant. Data in (B–H) are presented as mean ± SEM. Each dot indicates independent biological replicate.

To determine whether Iso-modulated microglia influence oligodendrocyte responses, we established a conditioned-medium paradigm (Figure 9A). Conditioned medium derived from Iso-treated microglia, but not direct Iso treatment, significantly increased GPR37 expression and ERK phosphorylation in oligodendrocytes subjected to OGD/R, indicating that Iso acts indirectly through microglial-derived signals (Figure 9B).

Functionally, conditioned medium from Iso-treated microglia or PSAP treatment markedly reduced oligodendrocyte apoptosis, whereas direct Iso treatment had no protective effect (Figure 9C). Notably, knockdown of *Gpr37* in oligodendrocytes abolished the protective effects of both PSAP and Iso-conditioned microglial supernatants (Figure 9D, E), demonstrating that GPR37 is required for microglia-to-oligodendrocyte signaling.

Together, these findings establish a causal mechanism in which Iso reprograms microglia in a PSAP-dependent manner, thereby promoting oligodendrocyte survival through GPR37–ERK signaling. This provides direct mechanistic evidence that Akk-derived metabolic signals drive white matter repair through a microglia-dependent intercellular pathway.

**Figure 9.**
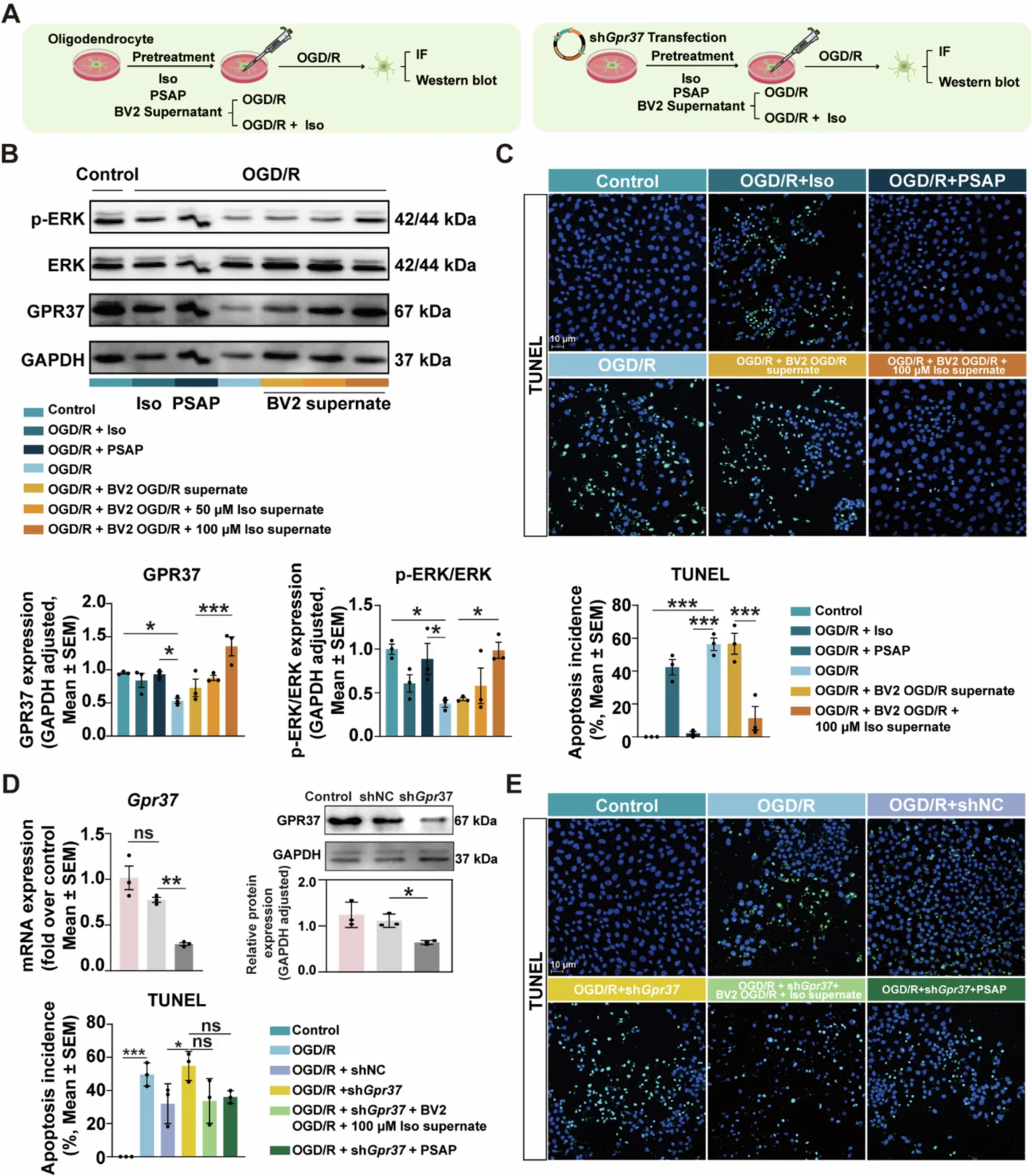
Iso–PSAP–GPR37 signaling mediates microglia–oligodendrocyte crosstalk. (A) Schematic of the experimental workflow, conditioned medium (CM) from microglia or Iso, PSAP was applied to oligodendrocytes or *Gpr37*-knockdown oligodendrocytes. (B) Western blot analysis of GPR37 expression and ERK phosphorylation in oligodendrocytes treated with conditioned medium from microglia or direct Iso or PSAP treatment. (C) TUNEL staining showing oligodendrocyte apoptosis under different treatment conditions. (D) the validation of *Gpr37* knockdown efficiency in oligodendrocytes using qRT-PCR and Western blot analyses, demonstrating reduced *Gpr37* mRNA and *GPR37* protein expression following *shGpr37* transfection. (E) representative TUNEL staining images and quantitative analysis of oligodendrocyte apoptosis under different treatment conditions, i llustrating the effects of *Gpr37* knockdown on oligodendrocyte survival in response to *PSAP* or conditioned medium from Iso-t reated microglia. (B–E): One-way ANOVA, followed by Holm modified Šidák post-hoc test. *p < 0.05, **p < 0.01, ***p < 0.001; ns, not significant. Data in (B–E) are presented as mean ± SEM. Each dot indicates independent biological replicate.

### GPR37 is required for Akk–Iso-mediated white matter repair

To determine whether GPR37 is required for the beneficial effects of Akk and Iso, we performed in vivo AAV-mediated *Gpr37* knockdown experiments (Figure 10A, B). *Gpr37* knockdown markedly attenuated Akk- and Iso-induced improvements in behavioral outcomes, including neurological and cognitive performance (Figure 10C, D). Consistently, *Gpr37* knockdown also reduced the improvement in corpus callosum white matter integrity and myelination induced by Akk and Iso (Figure 10E). These findings indicate that GPR37 is required for Akk/Iso-mediated functional recovery and white matter repair after ischemic stroke.

**Figure 10.**
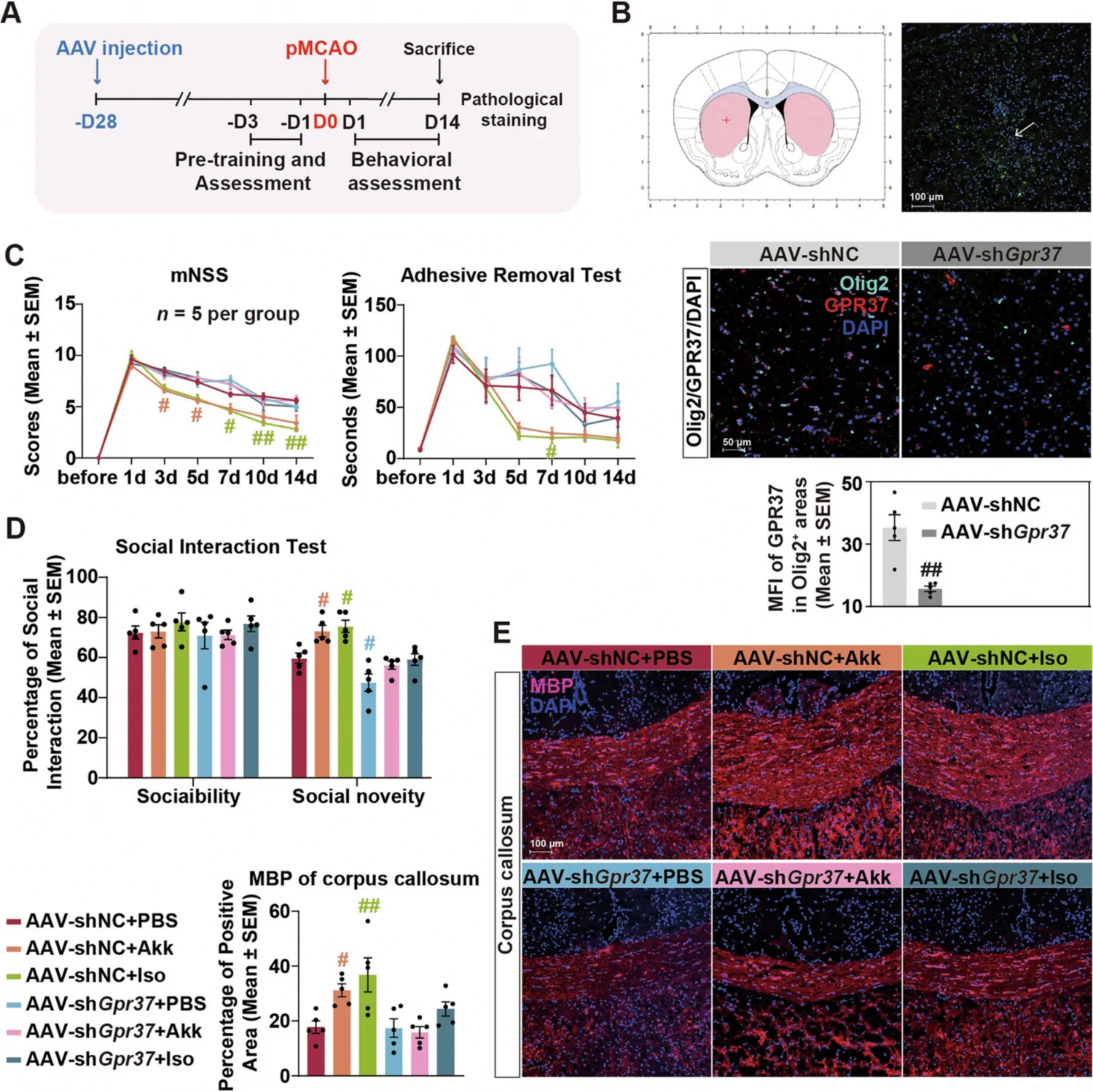
*Gpr37* knockdown attenuates the effects of Akk and Iso after IS. (A) Experimental timeline of AAV-mediated *Gpr37* knockdown, pMCAO induction, treatment, behavioral assessment, and tissue collection. (B) Stereotaxic injection site and representative images showing viral targeting and GPR37 expression in the striatum–corpus callosum region. (C) Behavioral assessment, including mNSS and adhesive removal test. (D) Cognitive/social behavioral assessment. (E) Immunofluorescence analysis of MBP in the corpus callosum. Intergroup differences were analyzed using one-way ANOVA followed by Holm–Šidák post hoc test. ^#^ *P* < 0.05, ^##^ *P* < 0.01 versus AAV-shNC + pMCAO + PBS group. Data are presented as mean ± SEM. Each dot represents an individual mouse.

## Discussion

Ischemic stroke (IS) results in persistent neurological deficits that extend beyond acute neuronal loss, with chronic neuroinflammation and white matter disruption posing major barriers to functional recovery.^19^ Although gut microbiota has emerged as an important modulator of stroke outcomes,^7^ ^,^ ^20^ how peripheral microbial signals are transduced into central nervous system repair remain poorly defined. Here, we define a microbiota–metabolite–glial signaling axis in which Akk promotes post-stroke recovery by driving white matter repair rather than acute neuroprotection. We identify Iso as a key circulating mediator and demonstrate that Akk-derived metabolic signaling reprograms microglia to promote oligodendrocyte maturation through PSAP–GPR37–ERK–dependent intercellular communication.

Importantly, *Akkermansia muciniphila* represents a consistent, core commensal component of intestinal microbiota,^21^ did not exert its effects through sustained expansion within the gut microbiota. Although Akk supplementation partially normalized microbial dysbiosis, modulation of individual commensal taxa—including restoration of *Turicibacter*-failed to reproduce the neuroprotective effects. These findings indicate that Akk-mediated benefits are unlikely to be explained solely by restoration of a stroke-depleted commensal taxon. Rather, Akk supplementation appears to promote functional and metabolic remodeling of the post-stroke gut ecosystem, highlighting circulating metabolites as key intermediaries linking gut microbial signals to brain repair.

At the cellular level, our data reveals microglial state reprogramming as a central mediator of Akk-associated recovery. ScRNA-seq revealed that emergence of lipid-laden, foamy microglial states following ischemic injury, characterized by dysregulated lipid metabolism and inflammatory activation, consistent with recent studies of lipid droplet–rich microglia.^22-25^ Akk supplementation reduced these maladaptive microglia states while restoring homeostatic and axon-tract–associated subsets. These changes were accompanied by reduced lipid droplet accumulation and attenuated inflammatory signaling, suggesting that Akk restores microglial lipid homeostasis and remodels the chronic post-ischemic inflammatory milieu.

Beyond microglia reprogramming, Akk treatment promoted oligodendrocyte lineage progression and white matter repair. Pseudotime analyses demonstrated accelerated differentiation of OPCs toward mature, myelinating phenotypes, accompanied by increased myelin basic protein expression in major white matter tracts. These findings position microglia–oligodendrocyte crosstalk as a key cellular mechanism through which microbiota-driven signals regulate post stroke repair.

Mechanistically, we identify the PSAP–GPR37–ERK axis as a molecular link coupling microglial reprogramming to oligodendrocyte maturation. Cell–cell communication analysis highlighted PSAP–GPR37 signaling as a prominently altered pathways following Akk treatment. Experimental validation confirmed increased PSAP production in microglia, upregulation of GPR37 in oligodendrocytes, and activation of downstream ERK signaling. These findings extend previous work on PSAP–GPR37 signaling in myelination and glial survival to the context of post-ischemic white matter repair.^26^ ^,^ ^27^ *In vivo* loss-of-function experiments further support the requirement of GPR37 signaling for Akk/Iso-mediated white matter repair after ischemic stroke.

Given the anatomical separation between the gut and brain, circulating metabolites likely serve as intermediaries linking Akk to central repair mechanisms. Untargeted metabolomics highlighted Iso as a prominent Akk-associated metabolite, whose plasma levels were correlated with functional recovery. Importantly, systemic Iso administration recapitulated major neurorestorative effects observed after Akk treatment, including suppression of microglial lipid accumulation, activation of PSAP–GPR37–ERK signaling, promotion of oligodendrocyte maturation, and enhancement of white matter integrity. These findings suggest that Iso contributes to Akk-associated neurorepair and functions as a potential systemic mediator linking peripheral metabolic changes with central repair mechanisms.

At the cellular level, Iso acted through a microglia-dependent mechanisms. Iso reprogrammed microglia toward a PSAP-secreting state, whereas direct Iso exposure had minimal effects on oligodendrocytes. Instead, conditioned medium from Iso-treated microglia activated GPR37–ERK signaling and enhanced oligodendrocyte survival under hypoxic stress. Critically, genetic knockdown of PSAP in microglia or GPR37 in oligodendrocytes markedly abolished these effects, demonstrating that PSAP–GPR37 signaling is required for Iso-mediated microglia–oligodendrocyte communication. These findings support a model in which Iso regulates microglial PSAP production and secretion, thereby promoting downstream GPR37–ERK signaling activation in oligodendrocytes and facilitating white matter repair.

Together, our findings define a previously unrecognized microbiota–metabolite–glial signaling axis that links peripheral microbial function to central nervous system repair. This study shifts the conceptual framework of microbiota–brain interactions from microbial composition toward metabolite-driven intercellular signaling, and identifies a therapeutically targetable pathway for promoting white matter repair after ischemic stroke.

### Limitations

Several limitations warrant consideration. First, this was a single-center observational study with a limited number of favorable outcome events. Despite multivariable adjustment and sensitivity analyses, residual confounding cannot be excluded and causal inference remains limited; larger, independent cohorts are required to validate the translational relevance of Akk and Iso. Second, only male mice were included to reduce experimental variability, and future studies incorporating both sexes are needed to assess potential sex-dependent effects. Third, although several taxa, including *Desulfovibrio*, *Pseudomonas*, *Streptococcus*, *Parasutterella*, and *Klebsiella*, were relatively enriched in patients without favorable early recovery, we did not test direct supplementation of these bacteria because sevral of them have been associated with dysbiosis, inflammation, or opportunistic pathogenicity.^28-33^ Future studies should determine whether targeted suppression or ecological replacement of these unfavorable-outcome-associated taxa can further improve post-stroke recovery. Finally, although exogenous Iso administration was sufficient to reproduce major beneficial effects of Akk treatment, direct loss-of-function approaches targeting Iso availability were not performed because specific and well-validated strategies to selectively deplete or inhibit Iso in vivo remain limited. Therefore, whether Iso is indispensable for all Akk-mediated effects remains to be determined. Future studies using Iso depletion strategies or pharmacological/genetic approaches targeting Iso metabolism will be required to establish its necessity in Akk-induced neurorepair.

In summary, our study defines a previously unrecognized microbiota–metabolite–glial signaling axis in which Akk drives post-stroke recovery through a repair-oriented mechanism rather than acute neuroprotection. We identify Iso as a key systemic mediator that reprograms microglia, activates PSAP–GPR37–ERK signaling, and promotes oligodendrocyte maturation and white matter repair after IS. By shifting the conceptual framework from microbial composition to metabolite-driven intercellular communication, our findings provide mechanistic insight into how gut-derived signals orchestrate central nervous system repair. Targeting this axis may represent a promising therapeutic strategy to enhance endogenous repair and functional recovery after stroke and other white matter–related neurological disorders.

## Supplementary Material

Supplementary Material 1012-2026_Supplementary_Methods_and_data.docx

## Data Availability

16S rDNA gene sequencing data: deposited in the NCBI Sequence Read Archive (SRA) under BioProject PRJNA1456286 (https://dataview.ncbi.nlm.nih.gov/object/PRJNA1456286?reviewer=8rd760a18k9eo8fahrsvt28rb1). ScRNA-seq data have been deposited in the NCBI Gene Expression Omnibus (GEO) under accession number GSE346377 (BioProject PRJNA1525225). and linked to BioProject PRJNA1456286. Metabolomics data: deposited in the MetaboLights database and cross-referenced with NCBI BioProject PRJNA1456286. All data will be made publicly available upon publication in accordance with the open data policy of Gut Microbes. In the meantime, the raw data are available from the corresponding author upon reasonable request for review purposes.
