# Supplementary material for: A microbiota–metabolite–glial axis drives white matter repair via isorhamnetin–PSAP–GPR37 signaling after ischemic stroke
Source: Gut Microbes. 2026 Nov 1;18(1):2748092. doi: 10.1080/19490976.2026.2748092 (PMC13743054; doi:10.1080/19490976.2026.2748092)
Supplement: Supplementary file 1 [file KGMI_A_2748092_SM4010.docx]

**Supplementary Methods and data**

**Baseline characteristics of study participants**

A total of 114 participants were included, comprising 28 healthy controls and 86 patients with ischemic stroke (IS). Detailed information regarding was presented in Supplementary Table S1. There were no significant differences between the two groups in sex, age, body mass index (BMI), or the prevalence of coronary heart disease, hyperlipidemia, smoking, or alcohol consumption. In contrast, IS patients had a significantly higher prevalence of hypertension, diabetes mellitus, and prior cerebrovascular disease compared with controls. These findings indicate that the IS cohort exhibited a higher burden of vascular risk factors, consistent with the clinical profile of stroke populations.

**Supplementary Table S1. Baseline Demographics and Clinical Characteristics of the Study Participants.**

| Variable | Overall  N = 114^1^ | Control  N = 28 (25%)^1^ | Ischemic stroke  N = 86 (75%)^1^ | statistics | p-value^2^ |
| --- | --- | --- | --- | --- | --- |
| Male (n) | 76 (66.67%) | 16 (57.14%) | 60 (69.77%) | 1.00 | 0.317 |
| Age (y) | 62.00 [57.00, 70.00] | 60.00 [54.75, 63.75] | 64.50 [58.00, 71.00] | 937.00 | 0.079 |
| BMI (kg/m^2^) | 25.71 [24.22, 27.02] | 25.99 [24.69, 28.62] | 25.33 [24.18, 26.88] | 1418.50 | 0.159 |
| Past Medical History (n) |  |  |  |  |  |
| Hypertension | 78 (68.42%) | 9 (32.14%) | 69 (80.23%) | 20.44 | <0.001 |
| Coronary heart disease | 18 (15.79%) | 3 (10.71%) | 15 (17.44%) |  | 0.555 |
| Diabetes | 39 (34.21%) | 4 (14.29%) | 35 (40.70%) |  | 0.012 |
| Hyperlipemia | 97 (85.09%) | 22 (78.57%) | 75 (87.21%) | 0.65 | 0.418 |
| Cerebrovascular disease | 35 (30.70%) | 2 (7.14%) | 33 (38.37%) |  | 0.002 |
| Smoking (n) | 63 (55.26%) | 11 (39.29%) | 52 (60.47%) |  | 0.082 |
| Drinking (n) | 54 (47.37%) | 13 (46.43%) | 41 (47.67%) | 0.00 | >0.999 |

^1^n (%); Median [IQR]

^2^Pearson's Chi-squared test; Wilcoxon rank sum test; Fisher's exact test

**Early short-term functional prognosis analysis**

After standardized treatment, the National Institutes of Health Stroke Scale (NIHSS) scores were evaluated. Compared with the admission NIHSS scores neurological outcomes varied among patient. Among the 86 IS patients, 15 were classified as having a favorable prognosis at discharge on 7 days, whereas 71 were categorized as having other outcomes. Baseline demographic characteristics, including age, sex, and BMI, vascular risk factors, stroke subtypes, antithrombotic treatment, gastrointestinal complications, and laboratory parameters, did not differ significantly between the two groups (Supplementary Table S2). These findings suggested that early neurological recovery was not explained by conventional clinical, or biochemical factors, supporting the potential involvement of additional nontraditional determinants. Given the limited number of patients with favorable prognosis, analyses were considered exploratory.

**Supplementary Table S2. Baseline characteristics of IS patients for 7-day prognosis.**

| Variable | Overall  N = 86^1^ | Favourable prognosis  N = 15 (17%)^1^ | Others  N = 71 (83%)^1^ | statistics | *p*-value^2^ |
| --- | --- | --- | --- | --- | --- |
| Male (n) | 60 (69.77%) | 12 (80.00%) | 48 (67.61%) |  | 0.537 |
| Age (y) | 64.50 [58.00, 71.00] | 66.00 [58.50, 69.50] | 64.00 [57.50, 72.00] | 567.00 | 0.698 |
| BMI (kg/m2) | 25.42 (2.06) | 25.44 (2.09) | 25.42 (2.07) | -0.03 | 0.973 |
| Past Medical History (n) |  |  |  |  |  |
| Hypertension | 69 (80.23%) | 12 (80.00%) | 57 (80.28%) |  | >0.999 |
| Coronary heart disease | 15 (17.44%) | 3 (20.00%) | 12 (16.90%) |  | 0.720 |
| Atrial fibrillation | 5 (5.81%) | 1 (6.67%) | 4 (5.63%) |  | >0.999 |
| Diabetes | 35 (40.70%) | 5 (33.33%) | 30 (42.25%) | 0.12 | 0.727 |
| Hyperlipemia | 75 (87.21%) | 14 (93.33%) | 61 (85.92%) |  | 0.680 |
| Cerebrovascular disease | 33 (38.37%) | 8 (53.33%) | 25 (35.21%) | 1.04 | 0.308 |
| Smoking (n) | 52 (60.47%) | 8 (53.33%) | 44 (61.97%) | 0.11 | 0.741 |
| Drinking (n) | 41 (47.67%) | 8 (53.33%) | 33 (46.48%) | 0.04 | 0.843 |
| Stroke subtypes (n) |  |  |  |  | 0.884 |
| Large artery atherosclerosis | 71 (82.56%) | 14 (93.33%) | 57 (80.28%) |  |  |
| Small vessel occlusion | 8 (9.30%) | 1 (6.67%) | 7 (9.86%) |  |  |
| Cardioembolism | 5 (5.81%) | 0 (0.00%) | 5 (7.04%) |  |  |
| Others | 2 (2.33%) | 0 (0.00%) | 2 (2.82%) |  |  |
| Dual antithrombotics (n) | 49 (56.98%) | 8 (53.33%) | 41 (57.75%) |  | 0.979 |
| Gastrointestinal complications (n) | 24 (27.91%) | 2 (13.33%) | 22 (30.99%) |  | 0.216 |
| Blood laboratory findings |  |  |  |  |  |
| Hemoglobin, g/L | 140.00 [125.25, 151.00] | 131.00 [125.00, 155.00] | 140.00 [125.50, 149.50] | 529 | 0.973 |
| WBC, ×10^9^/L | 6.75 [5.68, 8.11] | 7.20 [5.78, 8.16] | 6.71 [5.68, 8.03] | 504.5 | 0.754 |
| PLT, ×10^9^/L | 215.50 [184.00, 273.00] | 195.00 [169.50, 234.00] | 222.00 [188.50, 275.00] | 680.5 | 0.093 |
| Triglyceride, mmol/L | 1.42 [1.05, 2.30] | 1.50 [1.23, 2.31] | 1.39 [1.05, 2.28] | 501 | 0.724 |
| Total cholesterol, mmol/L | 4.71 (1.22) | 4.64 (0.86) | 4.73 (1.29) | 0.24 | 0.814 |
| Low density lipoprotein, mmol/L | 3.04 [2.40, 3.54] | 3.18 [2.56, 3.51] | 3.01 [2.34, 3.53] | 470 | 0.480 |
| High density lipoprotein, mmol/L | 1.12 [0.98, 1.39] | 1.12 [1.06, 1.66] | 1.11 [0.97, 1.37] | 404 | 0.145 |
| Creatine kinase, U/L | 72.00 [55.00, 96.25] | 70.00 [62.00, 94.50] | 73.00 [52.00, 95.50] | 485 | 0.593 |
| Creatinine, μmol/L | 68.00 [58.65, 80.30] | 69.60 [61.50, 72.80] | 68.00 [58.30, 81.15] | 521 | 0.900 |
| AST, U/L | 20.50 [18.00, 25.00] | 20.00 [16.50, 23.00] | 21.00 [18.00, 26.00] | 615 | 0.350 |
| ALT, U/L | 17.00 [14.00, 24.75] | 17.00 [14.00, 19.00] | 17.00 [14.00, 26.50] | 630.5 | 0.266 |

^1^n (%); Median [IQR]; Mean (SD)

^2^Fisher's exact test; Wilcoxon rank sum test; Pearson's Chi-squared test; Two Sample t-test

**Multivariable Firth penalized logistic regression analyses of the association between Akk abundance and favourable neurological recovery.**

To evaluate whether the observed association between Akk abundance and favourable neurological recovery was independent of clinically relevant confounders, multivariable Firth penalized logistic regression was performed. The primary model adjusted for age, sex, and dual antithrombotic therapy. Sensitivity analyses were conducted by additionally adjusting for hypertension, coronary heart disease, atrial fibrillation, diabetes, hyperlipemia, or previous cerebrovascular disease, one at a time (Supplementary Table S3).

**Supplementary Table S3. Multivariable and sensitivity analyses of the association between Akk abundance and favourable early neurological recovery.**

| Model | Adjustment | Adjusted OR (95%CI) | *P* value |
| --- | --- | --- | --- |
| Crude | None | 1.77 (1.11–2.82) | 0.017 |
| Model 1 | Age+Sex | 1.94 (1.19–3.28) | 0.008 |
| Primary | Age+Sex+Dual antithrombotics | 1.97 (1.20–3.35) | 0.008 |
| Sensitivity 1 | Primary+Hypertension | 1.96 (1.20–3.33) | 0.008 |
| Sensitivity 2 | Primary+CHD | 2.03 (1.23–3.49) | 0.006 |
| Sensitivity 3 | Primary+AF | 1.93 (1.19–3.25) | 0.008 |
| Sensitivity 4 | Primary+Diabetes | 1.99 (1.21–3.41) | 0.007 |
| Sensitivity 5 | Primary+Hyperlipemia | 1.92 (1.17–3.26) | 0.010 |
| Sensitivity 6 | Primary+CVD | 2.07 (1.25–3.60) | 0.005 |

Data are presented as adjusted odds ratios (ORs) with 95% confidence intervals (CIs) for each one-unit increase in log10-transformed Akk abundance. Multivariable analyses were performed using Firth's penalized logistic regression because of the limited number of favourable outcome events. Sensitivity analyses were conducted by adding one additional clinical covariate to the primary model individually.

**High-dose Akk alleviates intestinal inflammation and barrier dysfunction after pMCAO**

To assess the impact of IS on intestinal homeostasis and the therapeutic potential of Akk, intestinal tissues were collected at 14 days after pMCAO. Compared with sham-operated mice, PBS-treated pMCAO mice exhibited significant reduction in colonic length, indicative of intestinal injury. High-dose Akk treatment significantly restored colonic length, whereas low-dose Akk showed no significant effect (Supplementary Fig. S1A). Consistently, qRT-PCR analysis revealed that high-dose Akk supplementation significantly increased the anti-inflammatory cytokine *interleukin-10* (*Il10*) mRNA expression (Supplementary Fig. S1B). Histological examination of ileal and colonic tissues by hematoxylin and eosin (H&E) staining revealed inflammatory infiltration, epithelial disruption, crypt collapse, and a reduced villus height-to-crypt depth ratio. These pathological alterations were ameliorated in the H-Akk group (Supplementary Fig. S1C). In parallel, Alcian blue–periodic acid–Schiff staining revealed a reduction in mature goblet cell abundance after pMCAO, which was partially restored by H-Akk treatment (Supplementary Fig. S1D). Given the critical role of goblet cells in maintaining the intestinal mucus barrier, we further examined the expression of epithelial tight junction proteins. Western blot analysis showed that the levels of ZO-1 and Occludin were significantly downregulated in PBS-treated pMCAO mice and resorted by H-Akk (Supplementary Fig. S1E). Together, these findings indicate that Akk supplementation mitigates pMCAO-induced intestinal inflammation and barrier dysfunction.

**Supplementary Fig. S1.** **Therapeutic effects of Akk on intestinal inflammation and the damage of intestinal barrier after pMCAO.**

(A) Representative images and quantification of intestinal length in Sham, PBS-, low-dose Akk–, and high-dose Akk–treated mice at 14 days after pMCAO. (B) qRT-PCR analysis of IL-10 mRNA expression in intestinal tissues. (C) Representative hematoxylin and eosin–stained sections of ileum and colon, with quantification of villus height–to–crypt depth ratio in the ileum and histological injury scores in the colon. (D) Representative Alcian blue–periodic acid–Schiff–stained sections and quantification of mature goblet cells per crypt in the ileum and colon. (E) Representative Western blot images and densitometric analysis of tight junction proteins ZO-1 and Occludin in intestinal tissues. (A): Kruskal-Wallis H test, followed by Dunn’s post-hoc test for multiple comparisons. (B): Kruskal-Wallis H test, followed by Dunn’s post-hoc test for multiple comparisons. (C): One-way ANOVA for V/C analyze, followed by Holm modified Šidák post-hoc test for multiple comparisons; Kruskal-Wallis H test for Geboes scores analyze, followed by Dunn’s post-hoc test for multiple comparisons. (D): One-way ANOVA, followed by Holm modified Šidák post-hoc test for multiple comparisons. (E): One-way ANOVA for ZO-1 analyze, followed by Holm modified Šidák post-hoc test for multiple comparisons; Kruskal-Wallis H test for Occludin analyze, followed by Dunn’s post-hoc test for multiple comparisons. **p* < 0.05, ***p* < 0.01 vs Sham group; ^#^*p* < 0.05, ^##^*p* < 0.01, ^###^*p* < 0.001 vs PBS group; ns, not significant. Data in (A-D) are presented as mean ± SEM. Each dot indicates an individual mouse.

**High-dose Akk modulates post-stroke gut microbiota composition without increasing its own abundance**

16S rDNA sequencing revealed significant separation of gut microbial community structure among Sham, PBS-, and H-Akk–treated mice after pMCAO, as shown by principal coordinate analysis (PCoA, Supplementary Fig. S2A) and β-diversity metrics (Supplementary Fig. S2B). Genus-level community profiling demonstrated that Akk treatment partially normalized post-stroke dysbiosis toward a Sham-like microbial composition (Supplementary Fig. S2C). Consistent with the 16S rDNA sequencing data, Akk-specific qRT-PCR confirmed that *Akkermansia* was detectable in fecal samples from mice under our experimental conditions. Fecal Akk abundance showed a modest increase after pMCAO compared with sham controls. Notably, H-Akk supplementation did not increase fecal *Akkermansia* abundance (Supplementary Fig. S2D). Meanwhile, heatmap analysis increased abundance of several short-chain fatty acid–associated taxa *Lachnospiraceae-NK4A136* and *Bifidobacterium*. Specifically, the relative abundance of *Turicibacter* was significantly decreased in the PBS group but increased in the H-Akk group (Supplementary Fig. S2E), and the abundances of *Akkermansia* and *Turicibacter* are correlated (Supplementary Fig. S2F).

**Supplementary Fig. S2.** **High-dose Akk reshapes gut microbiota composition after pMCAO without increasing Akk abundance.**

(A) PCoA of gut microbial communities at the amplicon sequence variant (ASV) level in Sham, PBS-, and high-dose Akk–treated mice. (B) β-diversity analysis showing differences in microbial community dissimilarity among groups. (C) Genus-level community composition displayed as stacked bar plots. (D) Relative abundance of *Akkermansia* in fecal samples. (E) Heatmap of differentially abundant bacterial genera across experimental groups, highlighting *Turicibacter* and *Akkermansia*. (F) Correlation analysis between *Akkermansia* and *Turicibacter*. (A): Adonis (PERMANOVA) test. (B, D): Unpaired two-tailed Wilcoxon rank-sum test. (F): Spearman's rank correlation coefficient. ***p* < 0.01; ns, not significant. Data in (B, D) are presented as mean ± SD. Each dot represents an individual mouse, *n* = 6 for each group.

To test whether changes in individual taxa mediated the neuroprotective effects of Akk, *Turicibacter* was administered by gavage (Supplementary Fig. S3A), however, this intervention did not improve neurological outcomes after pMCAO (Supplementary Fig. S3B), indicating that the beneficial effects of H‑Akk are not attributable to secondary enrichment of *Turicibacter*.

**Supplementary Fig. S3. *Turicibacter* supplementation does not improve neurological function in pMCAO mice.**

(A) Schematic illustration of the experimental design. 6-8-month-old C57BL/6 mice were subjected to pMCAO. Mice were administered intragastrically with PBS, low-dose *Turicibacter* or high-dose *Turicibacter* from day 0 to day 14. A series of behavioural tests were performed. (B) Neurological outcomes following *Turicibacter* supplementation in pMCAO mice. (B): Intergroup differences at each time point were analyzed using One-way ANOVA, followed by Holm modified Šidák post-hoc test for normally distributed data; and Kruskal-Wallis H test, followed by Dunn’s post-hoc test for non-normally distributed data. ns, not significant. Data in (B) are presented as mean ± SEM.

**Supplementary Fig. S4. Detailed characterization of Akk-induced microglial and oligodendrocyte transcriptional remodeling after ischemic stroke.**

(A) Expression patterns of representative marker genes defining microglial subpopulations, including foam cells, ATM, DAM, and H-microglia. (B) Detailed ligand–receptor interaction network among major brain cell populations. (C) Expression profiles of oligodendrocyte lineage marker genes across six subclusters.

**Supplementary Table S4. Primers for qRT-PCR.**

| Gene | Forwards sequence (5’-3’) | Reverse sequence (5’-3’) |
| --- | --- | --- |
| *Tnfα* | TGCTCTGTGAAGGGAATGGG | ACCCTGAGCCATAATCCCCT |
| *Il1β* | AACCAGAGCACCCACTTACC | AGACTTCTGGGAAGTTCTACAGT |
| *Il6* | TGGTCTTCTGGAGTACCATAGC | TGACTCCAGCTTATCTCTTGGT |
| *Il10* | GCTCCAAGACCAAGGTGTCT | AGGACACCATAGCAAAGGGC |
| *Psap* | CCTGTCCAAGACCCGAAGAC | AAGGAAGGGATTTCGCTGTGG |
| *Gpr37* | AAGAATCCTACGGAGCCTA | TGAGAAAGTCCCAGAAAGC |
| *β-actin* | TGTACGTAGCCATCCAGGCT | TTCTCCAGGGAGGAAGAGGA |

**Akk does not activate the alternative PSAP–GPR37L1 signaling pathway.**

To determine whether Akk engages PSAP signaling pathways beyond microglia–astrocyte-oligodendrocyte axis, we first performed astrocyte activation by immunohistochemical staining for GFAP. No significant differences in astrogliosis were observed among Sham, PBS, and high-dose Akk groups (Supplementary Fig. S5A). We next examined the expression of the alternative PSAP receptor GPR37L1 in the ischemic brain. Neither *Gpr37l1* mRNA nor protein levels were significantly altered by high-dose Akk treatment compared with PBS controls (Supplementary Fig. S5B-D). These findings indicate that the benefit effect of Akk are not mediated through the PSAP–GPR37L1 axis.

**Supplementary Fig. S5.** **Limited effects of Akk on astrocyte activation and GPR37L1 expression in the ischemic brain.**

(A) Representative immunohistochemistry images of GFAP (astrocyte marker) staining in brain sections from Sham, PBS-treated, and H-Akk-treated groups. (B) *Gpr37l1* mRNA expression in brain tissues from Sham, PBS-treated, and H-Akk–treated mice. (C) Representative Western blot images and densitometric quantification of GPR37L1 protein expression. (D) Representative immunofluorescence images and quantitative analysis of GPR37L1 signal intensity in the peri-infarct region. (A): One-way ANOVA, followed by Holm modified Šidák post-hoc test for multiple comparisons. (B): Kruskal-Wallis H test, followed by Dunn’s post-hoc test for multiple comparisons. (C, D): One-way ANOVA, followed by Holm modified Šidák post-hoc test for multiple comparisons. ns, not significant. Data in (A-D) are presented as mean ± SEM. Each dot indicates an individual mouse.

**Specific sphingomyelin species are associated with better prognosis of neurological outcome.**

Several sphingomyelins (SM) species-including SM(d18:1/18:0), SM(d18:1/16:0), SM (d16:1/24:1(15Z)), SM(d17:1/24:1(15Z)), and SM(d17:1/24:0) were positively correlated with favorable prognosis (Supplementary Fig. S6).

**Supplementary Fig. S6. Spearman correlation analysis between SMs and neurobehavioral outcomes.**

**Effects of Iso and PSAP on cell viability in BV2 Microglia and Oligodendrocytes**

Based on the CCK-8 cell viability assay, Iso at concentrations of 200 μM and 500 μM significantly inhibited the viability of oligodendrocytes under OGD/R conditions. In contrast, isorhamnetin at 0–100 μM showed no cytotoxicity to BV2 microglia or oligodendrocytes in both normoxic and hypoxic environments (Supplementary Fig. S7A, B). Therefore, 50 μM and 100 μM isorhamnetin were chosen for all subsequent experiments. PSAP (1–1000 nM) exhibited no cytotoxicity to oligodendrocytes under either normoxic or OGD/R conditions. Thus, 1 μM PSAP was chosen for all subsequent in vitro studies (Supplementary Fig. S7C).

**Supplementary Fig. S7. Cytotoxicity screening of Iso and PSAP in BV2 microglia and oligodendrocytes.**

Cell viability was measured by CCK-8 assay, cells were treated with indicated concentrations of Iso (A, B) or PSAP (C) under normoxic or OGD/R conditions. Intergroup differences were analyzed using One-way ANOVA, followed by Holm modified Šidák post-hoc test; for normally distributed data and Kruskal-Wallis H test, followed by Dunn’s post-hoc test for non-normally distributed data, followed by Holm-Sidak post hoc test for pairwise comparisons. **p* < 0.05, ***p* < 0.01; ns, not significant. Data in (A-C) are presented as mean ± SEM. Each dot indicates a duplication.

**AAV-mediated *Gpr37* knockdown and stereotaxic injection**

Recombinant adeno-associated viruses (AAV9 serotype) encoding *Gpr37* shRNA (AAV9-*Gpr37*-RNAi) and corresponding negative control (CON688) were purchased from Genechem Technology (Shanghai, China). Viral titers were 3.81 × 10¹³ vg/mL and 2.24 × 10¹³ vg/mL, respectively.

Mice were anesthetized with isoflurane (3% for induction and 1.5% for maintenance) delivered in oxygen through a nose cone and placed in a stereotaxic apparatus. Adequate anesthetic depth was confirmed by the absence of the pedal withdrawal reflex, and body temperature was maintained at 37 ± 0.5°C using a thermostatically controlled heating pad throughout the procedure. A total volume of 1 μL viral suspension was unilaterally injected into the right striatum–corpus callosum region using a Hamilton microsyringe with a 26-gauge needle. The stereotaxic coordinates were as follows: anteroposterior (AP) +0.8 mm from bregma, mediolateral (ML) +1.6 mm, and dorsoventral (DV) −3.3 mm from dura.

Viral infusion was delivered at a rate of 0.1 μL/min. After infusion, the needle was left in place for 3 min to prevent reflux and was then slowly withdrawn. Mice were allowed to recover on a thermostatic heating pad.

Meloxicam (5 mg/kg) was administered subcutaneously once every 24 h for 2 days after the surgery as post-operative care for pain relief.

AAV injection was performed 4 weeks before pMCAO surgery to allow stable and sufficient knockdown of *Gpr37* expression. Subsequent Akk or Iso treatment, behavioral testing, and histological analyses were performed after stroke induction according to the experimental design.
